# AI-assisted models to predict chemotherapy drugs modified with C_60_ fullerene derivatives

**DOI:** 10.3762/bjnano.15.95

**Published:** 2024-09-19

**Authors:** Jonathan-Siu-Loong Robles-Hernández, Dora Iliana Medina, Katerin Aguirre-Hurtado, Marlene Bosquez, Roberto Salcedo, Alan Miralrio

**Affiliations:** 1 Tecnologico de Monterrey, Escuela de Ingeniería y Ciencias, Ave. Eugenio Garza Sada 2501, Monterrey 64849, Mexico; 2 Tecnologico de Monterrey, Institute of Advanced Materials for Sustainable Manufacturing, Monterrey 64849, Mexico; 3 Instituto de Investigaciones en Materiales, Universidad Nacional Autónoma de México, Circuito exterior s/n, Ciudad Universitaria, Coyoacán, 04510, Ciudad de México, Mexico

**Keywords:** breast cancer, CXCR7, drug nanocarriers, QSAR

## Abstract

Employing quantitative structure–activity relationship (QSAR)/ quantitative structure–property relationship (QSPR) models, this study explores the application of fullerene derivatives as nanocarriers for breast cancer chemotherapy drugs. Isolated drugs and two drug–fullerene complexes (i.e., drug–pristine C_60_ fullerene and drug–carboxyfullerene C_60_–COOH) were investigated with the protein CXCR7 as the molecular docking target. The research involved over 30 drugs and employed Pearson’s hard–soft acid–base theory and common QSAR/QSPR descriptors to build predictive models for the docking scores. Energetic descriptors were computed using quantum chemistry at the density functional-based tight binding DFTB3 level. The results indicate that drug–fullerene complexes interact more with CXCR7 than isolated drugs. Specific binding sites were identified, with varying locations for each drug complex. Predictive models, developed using multiple linear regression and IBM Watson artificial intelligence (AI), achieved mean absolute percentage errors below 12%, driven by AI-identified key variables. The predictive models included mainly quantitative descriptors collected from datasets as well as computed ones. In addition, a water-soluble fullerene was used to compare results obtained by DFTB3 with a conventional density functional theory approach. These findings promise to enhance breast cancer chemotherapy by leveraging fullerene-based drug nanocarriers.

## Introduction

Breast cancer is the most diagnosed cancer in women and the second leading cause of cancer-related mortality in women [1–2]. Heritage is the most critical risk factor, and 15 to 20% of breast cancer is familiar [3]. One of the characteristics of breast cancer is that it can be wholly cured given an early diagnosis [4]. The mortality rate from breast cancer has been reduced by 1.9% annually from 2002 to 2011 and 1.3% from 2011 to 2020 [5]. Diagnostics and treatments have continuously improved through the years. However, the situation is different in each country considering the costs and technological advances in each country. In the United States, 300,590 cases of breast cancer had been estimated for the year 2023, with a total of 43,700 deaths [6]. Latin America has over 210,000 new cases and around 60,000 deaths yearly [7]. For the year 2020, it was estimated that about 2.3 million breast cancer cases were diagnosed in women globally, and about 685,000 died from this disease [8]. A recurrent problem with standard treatments are the side effects. Regarding the use of chemotherapeutic drugs, such issues are nephrotoxicity of cisplatin, cardiotoxicity of doxorubicin, and pulmonary fibrosis from the use of bleomycin [9–11]. Besides, in the case of radiotherapy, fibrosis, atrophy, and neuronal damage caused by irradiation can occur [12–13]. Consequently, novel treatments try to reduce the secondary effects while retaining the benefits of standard approaches.

Chemotherapy is one of the most extensively applied treatments for breast cancer, with different drug targets depending on the type of cancer. Progesterone- or estrogen-receptor-positive tumors are related to cancers with low mortality [14]. Another common target in chemotherapy is human epidermal growth factor receptor 2 (HER2). Only 15 to 20% of all tumors are HER2-positive, overexpressing Erb-B2 receptor tyrosine kinase 2 (ERBB2) in the cell membrane. HER2 tumors are usually more aggressive than other ones, but the advantage is that their treatment is very effective [15]. Another chemotherapy target is the chemokine C-X-C motif receptor 7 (CXCR7) [16–17]. This G-protein is targeted because studies show a possible positive effect on inhibiting the metastasis of cervical cancer cells [18]. However, more clinical and preclinical studies on CXCR7 and its co-player CXCR4 are required since alterations have been detected in diseases such as cancer, central nervous system and cardiac disorders, and autoimmune diseases [16].

In recent years, nanomaterials have attracted the attention of different scientific communities by providing them with new solutions for drug delivery [19–20]. These nanotechnological applications have made it possible to obtain treatments that release substances at specific sites of interest, reducing the required drug amount and side effects. Nanostructures to form these drug delivery systems can be divided into organic and inorganic [19–20], with the latter one being the less extensively studied. One option currently considered in pharmacy and medicine is carbon-based nanomaterials because of their physicochemical, mechanical, electrical, thermal, and optical properties [19–20], as well as their capacity to modify existing drugs. Fullerene derivatives have been proposed recently, particularly those obtained from fullerene C_60_ [21]. The unmodified fullerene C_60_ is known as a “free radical sponge” because its double bonds tend to accept free radicals [22]. Because of its size, surface area, and capacity to extinguish or generate reactive oxygen species, C_60_ is very promising in medicine and clinical therapy [23–24]. It is also possible to modify pristine fullerenes by adding polar functional groups (e.g., –COOH, –OH, or –NH_2_), to improve water solubility, antioxidant properties, and even biological activity [25]. For instance, polyhydroxy fullerenes (PHFs) exhibit properties suitable for biomedical applications, such as water solubility, biodegradability, biocompatibility, and hypoallergic response. It has been shown that PHFs can inhibit cancer tumor growth and positively regulate the immune system [26]. The same is valid for carboxylated fullerenes [27]; for instance, C_60_[C(COOH)_2_]_3_ is well known for its high biological activity in plants [28] and within mitochondrial dynamics [29].

Since the evaluation of novel drugs is a task that requires significant human and material resources, innovative strategies have been formulated as alternatives. Quantitative structure–activity and quantitative structure–property relationships (QSAR/QSPR) are a paradigm that can be useful in choosing promising molecules, considering the information on inactive and active compounds, through in silico approaches. According to the QSAR/QSPR paradigm, a given activity/property, *f*, can be modeled using a set of quantitative descriptors, *x*_1_, *x*_2_, *x*_3_,..., *x_n_*, theoretically determined or measured by experiments [30]. A relationship *f*(*x*_1_, *x*_2_, *x*_3_,..., *x_n_*) can be defined to predict the activity or property of molecules after the evaluation of their quantitative descriptors. However, the QSAR/QSPR paradigm does not explain how to select the descriptors or how to build the mathematical function. Consequently, the following paragraphs discuss basic concepts about selecting descriptors and regression techniques implemented in this manuscript.

Lipinski’s rule of five is a compendium of guidelines commonly used to determine if a molecule can be proposed as an orally delivered drug according to its physicochemical properties. According to this rule, a drug compound should have a molecular weight below 500 g/mol, a octanol–water partition coefficient (LogP) below 5, less than five hydrogen bond donor sites, and less than ten hydrogen bond acceptors sites. It is possible to add two other conditions, namely polar surface area (PSA) ≤ 140 Å^2^ and less than ten rotatable bonds [31]. Taking advantage of the readiness of these quantities in public datasets, the current study proposes some of these quantities as potentially suitable descriptors for predictive models. Besides, Pearson’s hard–soft acid–base (HSAB) theory suggests other descriptors to describe and predict the interactions between chemical species, such as those between a drug molecule as a ligand and a protein [32]. These quantitative values are based on the vertical ionization energy (*I*) and electron affinity (*A*). According to Koopmans’ theorem, both can be approximated by *I* = −*E*_HOMO_ and *A* = −*E*_LUMO_, where *E*_HOMO_ is the energy of the highest occupied molecular orbital (HOMO), and *E*_LUMO_ is the energy of the lowest unoccupied molecular orbital (LUMO). It is advantageous to combine these properties to find out if an interaction between two species will occur and to obtain new quantitative relationships. Another helpful descriptor is the global electrophilicity, calculated as ω = χ^2^/2η [33]. Electrophilicity is related to the energetic stabilization that a species gains by obtaining an additional electron.

## Methods

First, 42 drugs related to chemotherapy treatments for breast cancer were proposed. Although the most notable fullerene derivatives for biological applications are those with several hydrophilic groups, the carboxylic acid derivative C_60_–COOH has been studied as well. Baglayan and coworkers carried out a conformation analysis within DFT to obtain the ground state structure for C_60_–COOH [34]. In addition, they discussed its usage as a potential drug carrier for the antimetabolic and anticancer drug 5-fluoruracil [34]. Similarly, Parlak and Alver reported a theoretical study on the interactions and stability of paracetamol complexes with C_60_–COOH [35]. Consequently, this work proposes the interaction of C_60_–COOH fullerene with anticancer drugs. As a complement, a water-soluble fullerene predicted as stable at the normal human body temperature was proposed to study the interactions with doxorubicin and gemcitabine [36]. The water-soluble fullerene is introduced to avoid known mutagenic reactions related to breast cancer [36]. It was also studied as a potential carrier for bedaquiline, an agent against tuberculosis [37]. The current study only considered molecules and complexes formed with up to 100 atoms to be affordable with our computational resources.

A set of descriptors was chosen to build the dataset, including molecular weight and p*K*_a_ [38]. Also, LogP was included, as a descriptor associated with the concentration of a given substance in the aqueous phase of a two-phase octanol–water mixture [39]. Similarly, LogS, related to the water solubility of a substance, was considered. Besides, PSA, as molecular surface associated with charge accumulation due to heteroatoms and polar groups, as well as polarizability (α) associated with the tendency of a given molecule to acquire an electric dipole moment in the presence of an external electric field were taken into account. The mentioned QSAR/QSPR descriptors were obtained from the Drugbank dataset (https://go.drugbank.com). Initial drug structures and connectivity were also obtained from the simplified molecular input line entry specification (SMILES) retrieved from Drugbank.

Molecular mechanics and density functional-based tight binding (DFTB) with dispersion and solvation corrections were used to obtain the optimized structures of the molecules under study and to compute *E*_HOMO_, *E*_LUMO_, and ω as quantitative descriptors. As an alternative to the most robust but computationally more expensive density functional theory (DFT) method, DFTB was used. A reference electron density ρ_0_ represents the sum of the neutral atomic densities [40]. Within the third-order approach DFTB3, the ground state density ρ(*r*) is obtained as the reference density ρ_0_ perturbed by density fluctuations δρ, that is,


[1] $$\begin{array}{c} E_{\text{DFTB}3}\left[\rho_{0}+\delta \rho \right]=E_{0}\left[\rho_{0}\right]+E_{1}\left[\rho_{0}+\delta \rho \right]\\ +E_{2}\left[\rho_{0}+{\left(\delta \rho \right)}^{2}\right]+E_{3}\left[\rho_{0}+{\left(\delta \rho \right)}^{3}\right]. \end{array}$$

For all calculations within DFTB3, the 3OB parameter set was used [41]. To carry out the global optimization procedure, Balloon 1.8.2 [42] and DFTB+ 17.1 [40] were used for the initial conformational study by genetic algorithms and final optimization at the DFTB3 level, respectively. London dispersion forces were considered in the DFTB3 and global optimization procedures by Lennard-Jones potentials, as implemented in UFF and MMFF94 force fields, respectively. The solvent effect was included by the Born solvation model within DFTB3. The study considered the chemotherapy drugs isolated and interacting with pristine C_60_ fullerene as well as its carboxylic acid derivative C_60_–COOH. Eight initial drug–fullerene structures were proposed to obtain their global optimization by means of DFTB3. The drugs were initially set at 1.5 Å of minimal distance from the fullerene. Once the global optimization was done, the same steps as for the isolated drugs were carried out for the molecular docking. The datasets were modified to take into account the effect of the fullerenes. Also, the validation set was reduced because of the large size of the complexes.

The atypical chemokine receptor 3, also known as CXCR7 or G-protein-coupled receptor 159 (GPR159) [16,18,43], was selected as the target protein for molecular docking. The iterative assembly refinement server (I-Tasser) was used to produce an initial structure for the CXCR7 protein by the homology approach. The sequence was extracted from the UniProtKB/Swiss-Prot dataset. From all homology structures produced by the I-Tasser server, the one with the highest confidence coefficients was selected to produce a reliable initial structure [44]. The lowest-energy structure, as in the study of Muthiah and coworkers [45], was validated using PROCHECK [46] to check the quality of the protein structure. The PDB produced with the previous step was subsequently optimized by an energy minimization through Amber force fields using the USCF Chimera 1.14 toolkit [47]. The secondary structural features were stabilized by TMpred [48] and HMMTOP [49] during energy minimization. Last, the protein was prepared by setting atomic charges and hydrogen atoms and merging the nonpolar groups. Once the structures were optimized, molecular docking was performed with the CXCR7 protein, using Autodock Vina 1.1, to obtain the docking score, established hydrogen bonds, and the binding site (pocket). The above was done for all drugs in the dataset and an external validation set.

IBM Watson AI was used to build the models and to predict the docking score through the Extra Trees regressor algorithm [50–51]. It was also used to obtain the most significant quantum descriptors used in each model. Extra Trees, an abbreviation of “extremely randomized trees”, is a mathematical method used to estimate a relationship between data and the covariates [52]. The Extra Trees algorithm creates many decision trees [52], but the sampling of each one is random. Thus, a dataset for each tree contains unique samples. The optimization of the hyperparameters associated with the decision trees obtained was performed by the derivative-free global search algorithm known as RBfOpt, which fits a radial basis function mode to accelerate the discovery of the hyperparameters [53]. All the above was used through the AutoAI tool within IBM Watson, an automatized routine to select the model with the best performance among those available in the platform. Since this method does not produce exportable mathematical models, another approach was used as detailed below [50].

Multiple linear regression (MLR) could be a tool to solve the problem in a complementary way to Extra Trees regression. MLR is a mathematical model that can be seen as an extension of linear regression. In terms of *n* input variables, *x*_1_, *x*_2_,…, *x_n_*, the outcome *y* can be expanded by the following linear expansion [54]:


[2] $$y=\beta_{0}+\beta_{1}\times x_{1}+\beta_{2}\times x_{2}+\beta_{3}\times x_{3}+...+\beta_{n}\times x_{n}.$$

In Equation 2, β*_k_* are the partial regression coefficients, and β_0_ is the value of *y* when all variables are set to zero.

To obtain the AI and MLR models, a fivefold approach was implemented, by using 80% of the data available to obtain the predictive model as training set and the remaining 20% as testing set. Supporting Information File 1 gives the results of the cross-validation for all the models reported in the current manuscript. Once the models were built, an additional external validation set was used to obtain evaluation metrics and to determine the most accurate models between methodologies. The metrics proposed to evaluate the performance of the predictive models were mean squared error (MSE), mean absolute percentage error (MAPE), mean absolute error (MAE), and root mean squared error (RMSE). These metrics were computed as follows:


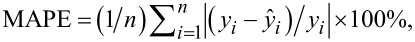



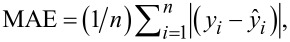



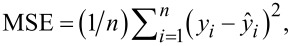


and


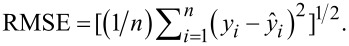


**Figure 1:**
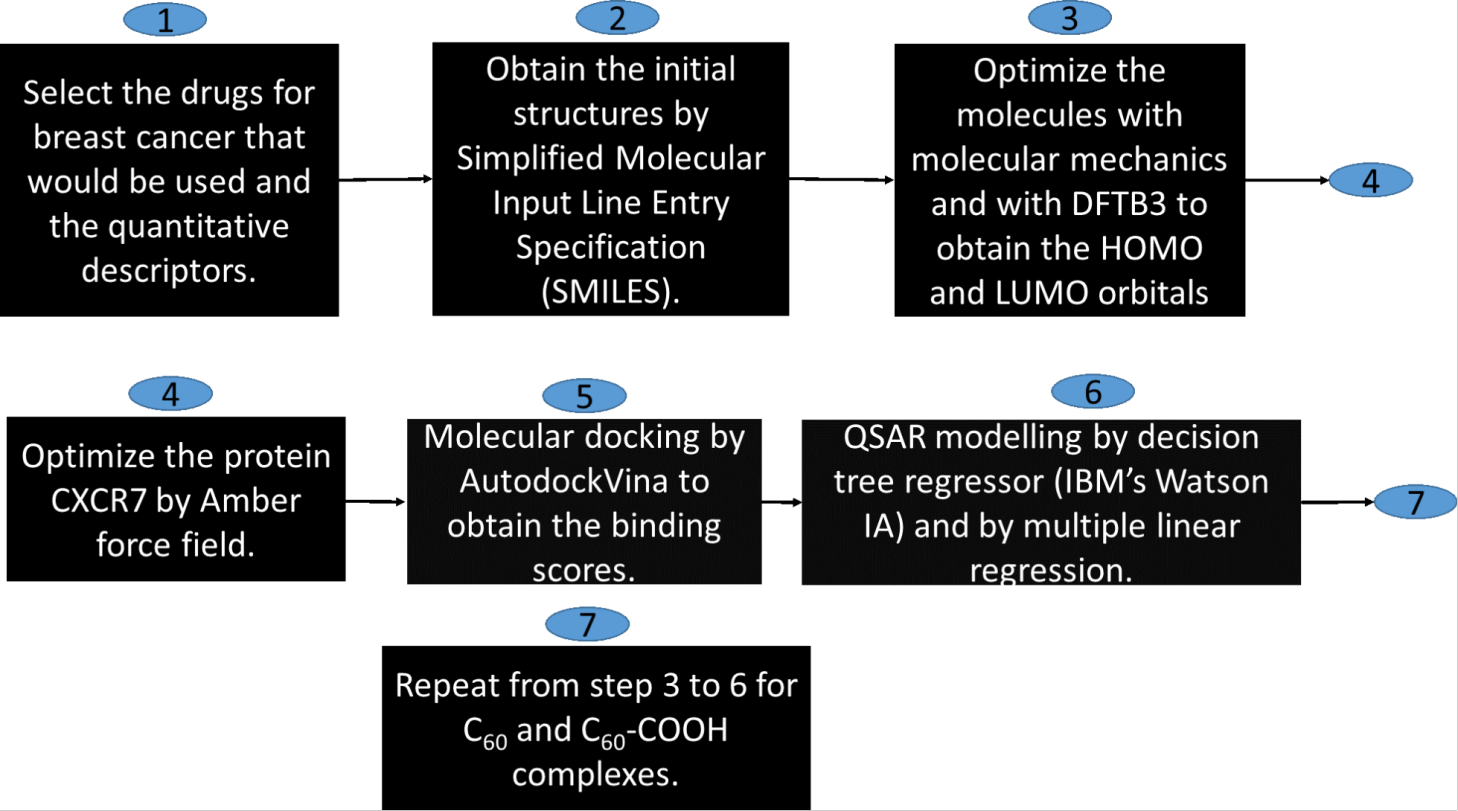
Workflow diagram of the stages during modeling.

Here, *y_i_* is the docking score for compound *I*, *ŷ_i_* is the estimated value of the docking score for compound *I* provided by the model. The workflow diagram in Figure 1 summarizes the procedure followed to obtain the models.

## Results and Discussion

Table 1 presents the quantum descriptors proposed for the current study and the symbols used for them. The physical unit of each descriptor, as well as references to their usage in similar QSAR/QSPR models, were included as well.

**Table 1:** Quantitative descriptors proposed to model the docking score of the isolated drugs, as well as of those modified with fullerenes C_60_ and C_60_–COOH, interacting with the protein CXCR7.

Variable	Descriptor	Symbol	Unit	Reference

*x*_1_	molecular weight	MW	g/mol	[55–56]
*x*_2_	water solubility	WS	mg/mL	[57]
*x*_3_	octanol–water partition coefficient	LogP	—	[58–59]
*x*_4_	solubility coefficient	LogS	—	—
*x*_5_	acid dissociation constant	p*K*_a_	—	[60]
*x*_6_	hydrogen acceptor count	Ac	—	[61]
*x*_7_	hydrogen donor count	Dn	—	[61]
*x*_8_	polar surface area	PSA	Å^2^	[62]
*x*_9_	rotatable bond count	RBC	—	[63]
*x*_10_	polarizability	α	Å^3^	[64]
*x*_11_	number of rings	NOR	—	[65]
*x*_12_	energy of HOMO	*E*_HOMO_	eV	[66–67]
*x*_13_	energy of LUMO	*E*_LUMO_	eV	[66–67]
*x*_14_	electrophilicity	ω	eV	[66–67]

### Isolated drugs

A dataset containing all the descriptors of Table 1 for 33 drugs was created to obtain the predictive models. Also, another nine compounds were considered to build an external validation set, allowing for the comparison between methodologies (Supporting Information File 1, Table S1). In the case of the training set, the molecular weight was obtained with values between 130.08 and 915.4 g/mol. Water solubility values varied between 0.0004 mg/mL and 22.3 mg/mL. The LogP values varied between −2 and 6.54, whereas LogS ranged from −6 to −1.1. Besides, p*K*_a_ values varied between −8 and 14.55. The hydrogen acceptor count varied widely between 2 and 13, whereas the hydrogen donor count varied between 0 and 6. In addition, the polar surface area had variations between 12.47 and 221.29 Å^2^. The cases of thiotepa and aldoxorubicin were not considered because they are part of the validation set. Rotatable bonds were obtained ranging from 0 to 15. The polarizability varied from 9.46 to 87.46 Å^3^; everolimus was excluded as part of the external validation set. Also, values of number of rings were obtained from 0 to 9. The energy of the HOMO was computed ranging from −7.400 to −4.392 eV, and the LUMO energy from −5.341 to −0.889 eV. Finally, the electrophilicity varied from 2.12 to 180.39 eV. Figure S1 (Supporting Information File 1) shows the correlation matrix between the ten most relevant quantum descriptors used to obtain the mathematical models. There are significant correlations between the molecular weight and the polarizability of about 0.93 and between polarizability and the number of rings of about 0.88. Also, molecular weight and number of rings, as well as WS and LogS exhibited considerable correlations, with values of 0.87 and 0.73, respectively. However, all variables showed a correlation below 0.95. Once the drugs were optimized, blind molecular docking was performed with the CXCR7 protein to obtain the docking score, number of established hydrogen bonds, and the protein residues interacting with the ligands in a coordination sphere of 3 Å. The results obtained with Autodock Vina [47,68] for training–testing and validation sets are shown in Table 2.

**Table 2:** Docking score, number of established H-bonds, and protein–ligand interacting residue in three-letter symbol, up to 3 Å distance. Drugs marked with an asterisk were used as external validation set.

Ligand	Docking score (kcal/mol)	H-bonds	Interacting residues

doxorubicin	−8.4	5	Asp275, Phe294, Leu297, Tyr200, His298, Arg197, Ser198, Cys196, Asp179, Trp100
neratinib maleate	−8.5	2	Ile276, Leu297, Phe294, His298, Tyr 268, Gln30, Ser216, Ser198, Phe129, Asp179, Leu183, His121, Phe124
epirubicin	−8.1	2	Asp275, Leu297, Tyr200, Ser198, Arg197, Cys196, His121, Trp100, Leu104, His298, Phe294, Gln301,
lapatinib ditosylate	−8.9	1	Glu213, Ile276, Val272, Leu297, Tyr200, His298, Arg197, Ser198, Leu183, His121, Cys196,
fulvestrant	−7.9	0	Leu297, Ile276 Val272, Tyr268, Gln301, Trp100, Phe124, Cys196, Asp179, Ser198, Ser216, Arg197
dinaciclib	−8.4	1	Leu297, Gln301, Tyr268, Trp100, Cys196, Arg197, Phe124, Leu128, Ser198, Asp179, Phe129, Met212, Ser216
abemaciclib	−9.8	1	Asn108, Trp100, Trp110, Leu104, Arg197, Lys40, Asn36, Pro38, Leu43, His298
gemcitabine	−6.2	0	Asn36, Phe294, Met37, Pro38, Asn39, Leu43, Lys40, Leu104, Asn108
voruciclib	−8.8	0	Asn36, Pro38, Asn39, His298, Leu43, Lys40, Leu104, Asn108, Trp100, Ser103, Trp110, Arg197
fluorouracil	−4.6	1	Asn319, Asn321, Lys73, Ile83, His80
letrozole	−8.1	0	His298, Gln301, Arg197, Leu104, Trp100, Ser198, His121, Trp110, Leu183
olaparib	−10.1	2	Tyr268, Val272, Ile276, Glu213, Leu209, Ile205, Met212, Tyr200, Phe199, Ser198, Leu128, Phe124
paclitaxel	−9.6	0	His298, Phe294, Leu297, Tyr268, Gln301, Val272, Leu104, Trp100, Phe124, His121, Trp110, Arg197, Ser198, Leu209, Tyr200, Leu183
seliciclib	−7.6	0	Leu183, Asp179, Ser125, Phe124, Phe129, Leu128, Ser216, Tyr268, Leu297
ixabepilone	−8.2	0	Leu297, Val272, Tyr268, Trp265, Phe124, Arg197, His298, Leu128, Ser198, Leu209, Tyr200, Met212, Ser216
anastrozole	−7.7	0	Met212, Leu209, Ser216, Tyr200, Phe129, Asp179, Ser125, Ser198, Phe124, Leu183, His121
pentostatin	−6.3	3	Phe294, Lys40, Leu104, Trp100, Asn108, Trp110
alvocidib	−8.6	1	His298, Leu43, Lys40, Leu104, Trp100, Asn108, Ser103, Trp110, Arg197
methotrexate	−8.5	3	Leu104, Gln301, Tyr268, Val272, Trp100, Trp110, Cys196, Phe124, His121, Ser198, Leu183, Asp179, Phe129, Glu213, Ser216
milciclib	−9.2	0	Lys40, Phe294, Leu297, Leu104, Asn108, Tyr268, Arg197, Val272, His121
ribociclib	−9.5	1	Lys40, Leu104, Leu43, Tyr195, Cys196, Arg197, Phe124, His121, Ser198, Leu183, Asp179
exemestane	−8.0	0	Phe294, Leu43, Lys40, Leu104, Trp110
tamoxifen	−7.9	1	Asn39, Pro38, Leu43, Lys40, Leu104, Trp110, Tyr195, Arg197
idarubicin	−8.8	1	Phe294, His298, Leu43, Leu104, Asn108, Ser103, Trp110, Cys196, His121
palbociclib	−8.3	1	Phe294, Asn36, Pro38, Leu43, Lys40, Leu104, Asn108, Arg197, Cys196
toremifene	−7.4	0	Leu43, Asn39, Leu104, Pro38, Lys40, Asn36, Phe294, Trp110, Tyr195, Arg197
vinblastine	−9.5	2	Leu43, Asn39, Leu104, His298, Phe294, Lys40, Asn108, Trp110, Arg197, Tyr195, Ile205, Leu209
pirarubicin	−9.4	1	Val272, Leu297, Tyr268, Gln301, Met212, Ile205, Leu209, Glu213, Arg197, Phe199, Trp110, Cys196, Phe199,
roniciclib	−8.4	1	Asn39, Met37, His298, Phe294, Pro38, Asn36, Trp100, Leu104, Lys40, Ser103, Asn108, Trp110,
capecitabine	−7.7	2	Gln301, Phe129, Phe124, Ser198, Arg197, His121, Cys196, Trp110, Leu183
sulfanilamide	−5.4	2	Tyr268, Val272, Glu213, Ser216, Leu128, Phe129, Met212, Ser125
zoledronic acid hydrate	−5.7	1	Tyr268, Glu213, Ser216, Phe129, Phe124, Ser125, Ser198, Met212, Asp179
pamidronic acid	−4.8	4	Val272, Tyr268, Trp265, Val217, Glu213, Ser216, Val215, Phe129, Met212, Val175, Phe124, Ser125, His121, Ser76, Tyr200, Asp179
docetaxel*	−9.5	2	Leu209, Glu213, Met212, Ser198, Ser216, Arg197, Leu183, Phe124, His121, Trp110, Ile276, Val272, Leu297, Tyr268, His298, Gln301, Leu104
topotecan*	−8.2	2	Tyr268, His298, Gln301, Trp100, Leu104, Trp110, Cys196, Phe124, Leu183, Ser198
tucatinib*	−10.1	1	Val272, Tyr268, Leu104, Arg197, Ser198, Trp110, His121, Phe124, Asp179, Leu183
aldoxorubicin*	−8.4	1	Ile205, Leu209, Tyr200, Ser198, Asp179, Arg197, Leu193, Asp179, His121, Phe124, Ser125, Trp110, Asn108, Leu104, His298, Gln301, Leu297, Val272, Tyr268
vinorelbine*	−9.1	0	Leu297, Tyr268, Phe294, His298, Gln301, Leu43, Leu47, Leu104, Arg197, Trp110, Ser198, Tyr200, Leu209
etoposide*	−9.3	2	Lys 40, Leu43, Asn108, Leu104, Trp100, Trp110, His121, Phe124, Leu183, Gln301
pemetrexed*	−9.1	2	Leu104, Ser103, Trp100, Trp110, Cys196, His121, Phe124, Leu183, Ser198, Tyr200, Val272, Glu213, Ser216
everolimus*	−9.2	3	Leu209, Met33, Asn36, Phe294, His298, Asn39, Leu43, Lys40, Leu104, Asn108, Tyr195, Arg197
thiotepa*	−3.9	1	Tyr268, Val272, Ser216, Phe124

The docking scores ranged from −10.1 to −4.6 kcal/mol for the training set. The molecule with the most significant bond strength, according to its docking score, was olaparib, whereas the one with the lowest bond strength was fluorouracil. The number of hydrogen bonds was computed ranging from 0 to 5. It is important to note that the number of hydrogen bonds is not directly related to the docking score since there are weak and strong hydrogen bonds. This assumption was proved by the analysis performed to obtain the predictive models, as discussed below.

**Figure 2:**
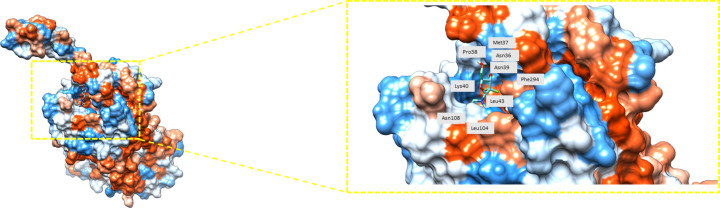
Gemcitabine after blind docking with the protein CXCR7. Blue color indicates the most hydrophilic sites and orange-red the most hydrophobic ones. Interacting residues are labeled as well.

According to the results of the interacting residues annotated in Table 2, two things can be highlighted. First, the analyzed isolated drugs bind inside the protein CXCR7 (Figure 2); second, the pocket is similar for several analyzed drugs. For example, the leucine residue Leu297 is shared by eleven drugs, indicating that their binding zone is close to each other. Comparing the interacting residues with those recently obtained by Muthiah et al. [45], it is possible to conclude that the pockets are similar. For instance, doxorubicin was obtained in both cases with Asp179, Cys196, and Trp100 as interacting residues. Also, similar pockets could be obtained because the selected drugs are mostly designed to serve as chemotherapy agents for breast cancer. Thus, it is possible to assume that several drugs share a common mechanism of action and, subsequently, a common protein target, such as CXCR7. For instance, gemcitabine, shown in Figure 2, shares the pocket within CXCR7 with several drugs.

The quantitative descriptors included in the produced models are discussed next. Table 3 contains the used quantitative descriptors and the importance that each one has in the mathematical models. The docking score was the predicted variable in all cases. In addition, the correlation matrix (Supporting Information File 1, Figure S1) was used to build the models. Among the IBM Watson AI models, the first one was obtained using all the initially proposed quantitative descriptors (Table 1). In the second and fourth models, polarizability was not used because, as shown in the correlation matrix, it was found to be closely related to molecular weight. The third and fifth models did not consider molecular weight because of the same relationship with polarizability. Also, the descriptors p*K*_a_, Ac, and PSA were not considered because their importance in the previous models was below 10% (Table 3). Although conceptually different, *E*_HOMO_ was discarded because it is related to *E*_LUMO_ and electrophilicity. The last model did not include α because of its relationship with MW. It is important to notice that the most important variables in the six models were NOR, polarizability, LogS, MW, and WS. The least important variables in the six models were *E*_LUMO_, p*K*_a_, PSA, *E*_HOMO_, and Ac; they all had less than 10% importance in all models. Hence, the computed *E*_HOMO_ and *E*_LUMO_ values were not particularly useful in predicting the ligand–protein docking score and, subsequently, the docking score.

**Table 3:** Input variables (IV) and output importance (OI) of six Extra Tree regressor models obtained from IBM Watson. Variables are annotated according to Table 1. The best model, according to the MAPE values, is highlighted in bold.

Model 1		Model 2		Model 3		Model 4		Model 5		Model 6	
					
IV	OI (%)	IV	OI (%)	IV	OI (%)	IV	OI (%)	IV	OI (%)	IV	OI (%)

NOR	100	WS	100	**WS**	**100**	LogS	100	NOR	100	LogS	100
α	57	NOR	97	**LogS**	**99**	MW	53	α	74	NOR	15
LogS	43	LogP	94	**α**	**56**	NOR	26	LogS	49	*E*_LUMO_	7
WS	38	LogS	87	**NOR**	**39**	WS	17	WS	31	MW	6
MW	33	MW	20	**ω**	**18**	ω	12	LogP	19	ω	5
LogP	32	Dn	14	***E*** **_LUMO_**	**6**	*E*_LUMO_	5	Dn	13	RBC	0
Ac	8	RBC	12	***E*** **_HOMO_**	**5**	*E*_HOMO_	3	*E*_LUMO_	5	Dn	0
Dn	6	*E*_LUMO_	7	**PSA**	**4**	PSA	3	RBC	0	Ws	0
*E*_HOMO_	2	PSA	4	**p** ***K*** **_a_**	**1**	p*K*_a_	1	ω	0	LogP	0
RBC	1	*E*_HOMO_	3	**LogP**	**1**	RBC	0	—	—	—	—
PSA	1	p*K*_a_	2	**RBC**	**0**	Ac	0	—	—	—	—
ω	1	Ac	1	**Dn**	**0**	Dn	0	—	—	—	—
p*K*_a_	0	Ω	0	**Ac**	**0**	LogP	0	—	—	—	—
*E*_LUMO_	0	—	—	—	—	—	—	—	—	—	—

To compare the performance offered by the Extra Trees algorithm of Watson AI, a comparison with a family of MLR models was made. Supporting Information File 1 contains the cross-validation for all reported MLR models. Table S2 (Supporting Information File 1) shows the docking scores obtained for the validation set by using both methodologies. With these values, it is possible to appreciate the difference between the docking score obtained directly by molecular docking through Autodock Vina and the prediction of the mathematical models for the external validation set.

To clearly state the performance comparison between AI and MLR, Table 4 reports the values computed for the evaluation metrics proposed for each model. The MSE ranged from 0.30 to 1.73 kcal^2^/mol^2^, whereas the MAPE varied from 6.1 to 16.37%. Also, MAE values from 0.46 to 1.13 kcal/mol and RMSE values from 0.55 to 1.32 kcal/mol were obtained. The above shows that both AI and MLR approaches accurately model the protein–ligand docking score, yielding higher confidence in the case of Extra Tree regressor models. The best performance, according to the computed minimum errors, was obtained in the case of Watson AI model 3. In contrast, the maximum error was obtained in MLR model 2. Thus, the variables denoted in this work can be used for other authors to propose novel chemotherapy drugs assuming CXCR7 as a target.

**Table 4:** Comparison metrics obtained by the use of AI and MLR in the case of isolated drugs. The best model, according to the MAPE values, is highlighted in bold. The values were computed relative to the validation set.

Error	Model 1		Model 2		Model 3		Model 4		Model 5		Model 6	

	AI	MLR	AI	MLR	AI	MLR	AI	MLR	AI	MLR	AI	MLR

MSE (kcal^2^/mol^2^)	0.93	1.69	0.64	1.73	**0.30**	**1.02**	0.43	1.51	0.82	1.37	0.73	1.10
MAPE (%)	11.51	16.11	9.65	16.37	**6.17**	**11.98**	6.70	15.69	10.92	14.10	10.34	12.49
MAE (kcal/mol)	0.77	1.11	0.64	1.13	**0.46**	**0.82**	0.51	1.08	0.71	0.98	0.66	0.83
RMSE (kcal/mol)	0.97	1.30	0.80	1.32	**0.55**	**1.01**	0.66	1.23	0.91	1.17	0.85	1.05

As mentioned above, the best model was AI model 3 with a MAPE of about 6.17%. Since the AI models are not exportable, our best model is represented by the following functional form: AI3_DRUG_ = *f*(WS, LogS, α, NOR, ω, *E*_LUMO_, *E*_HOMO_, PSA, p*K*_a_, LogP). The model with the lowest MAPE among those obtained by MLR, computed as 11.98%, is model 3, represented as follows:


[3] $$\begin{array}{c} \text{MLR}3_{\text{DRUG}}=-5.288+0.049\times \text{WS}+0.318\times \text{LogS}\\ +0.048\times \alpha -0.637\times \text{NOR}+0.009\times \omega \\ -0.054\times E_{\text{HOMO}}+0.113\times E_{\text{LUMO}}\\ -0.008\times \text{PSA}-0.234\times \text{LogP}-0.014\times \text{p}K_{\text{a}}. \end{array}$$

Thus, the AI was useful in selecting the most relevant variables for the formulation of the linear, accurate, and exportable model. Thus, AI is a valuable guide to obtain mathematical models with other methodologies such as MLR. Another significant model is MLR model 6 with the second lowest MAPE of 12.49%. It is worth noting that the model is compact and includes the computed descriptors E_HOMO_ and ω, as well as experimentally determined ones.


[4] $$\begin{array}{c} {\text{MLR6}}_{\text{DRUG}}=-4.2179+0.4131\times \text{LogS}-0.5274\times \text{NOR}\\ +0.2869\times E_{\text{HOMO}}+0.0009\times \text{MW}\\ +0.0088\times \omega . \end{array}$$

Although the best AI model was model 3, model 4 is relevant as well. It has the following functional form: AI4_DRUG_ = *f*(LogS, MW, NOR, WS, ω, *E*_LUMO_, *E*_HOMO_, PSA, p*K*_a_). For this model, the MAPE is about 6.70%. The variables used can be mostly evaluated by computational methods, except for p*K*_a_ and LogS.

### Drugs modified with C_60_

Since this study aims to elucidate the potential use of AI suites, such as Watson, to predict the docking score of pristine and modified chemotherapy drugs, the following paragraphs detail the extension of our datasets and models to drugs modified with potential nanocarriers. First, a dataset with 28 drugs, extracted from public datasets or modified from the data annotated in the previous case, was built with the corresponding quantitative descriptors to study complexes of the drugs with fullerene C_60_ or a simple C_60_–COOH derivative [29]. The resultant dataset is shown in Supporting Information File 1, Table S3. Complexes of the drugs with C_60_ or its derivative with more than one hundred atoms were excluded to save computational resources. Because of that, some maximums and minimums were modified in the dataset. Also, quantities such as the molecular weight or number of rings were shifted to the correspondent values for the drug–fullerene complexes since these modifications are only additive constants. Molecules interacting with C_60_ were studied in this subsection and those modified with the fullerene derivative are the described in the subsequent subsection.

In case of the complexes with C_60_, the molecular weight is obtained with values above 907.1639 g/mol. The above imposes some restrictions on the usage of fullerene derivatives as drug nanocarriers, since it is accepted that common pharmacological agents applied in topical therapies are under 500 Da [69]. For simplicity, WS, LogP, LogS, p*K*_a_, and α values were taken from isolated drugs. In the case of Dn, Ac, RBC, and PSA, values of the isolated drugs were also used. This is because fullerene is not expected to modify these descriptors since its chemical constitution lacks polar groups or donor/acceptor atoms. The number of rings was only modified with the addition of the fullerene rings from 32 to 39.

As stated in the Methods section, HSAB descriptors were computed for the drug–C_60_ complexes in their ground states while drugs with C_60_ were structurally optimized at the DFTB3 level. Since introducing a species known to act as an electron acceptor, such as fullerene C_60_, could modify the electron structure of the modified species [70–71], the energies of the frontier orbitals were recomputed for the modified drugs in their ground states. The energy of the HOMO varied between −3.458 and −5.718 eV. The energy of LUMO ranged from −3.179 to −5.388 eV. The electrophilicity, computed through Koopman’s theorem, varied from −134.88 to 280.8 eV. Once the drugs with fullerene C_60_ were globally optimized, molecular docking was conducted with the CXCR7 protein to acquire the docking score, number of established hydrogen bonds, and protein residues interacting with the complex at a distance of 3 Å. The results obtained with Autodock Vina for the 24 complexes and the validation set are presented in Table 5.

**Table 5:** Docking score, number of established H-bonds after docking, and protein–ligand interacting residues up to 3 Å distance obtained for the drugs modified with C_60_ fullerene. Drugs with the asterisk were used as an external validation set.

Ligands	Score (kcal/mol)	H-bonds	Interacting residues

doxorubicin	−9.2	2	Arg323, Ser316, Val313, Leu326, Phe330, Leu340, Leu343, Val64, Ile57, Ile60
epirubicin	−9.2	0	Arg323, Ser316, Leu326, Tyr322, Leu340, Leu343, Lys342, Val64, Pro312, Ala61, Ile60
lapatinib ditosylate	−9.4	0	Arg323, Ser316, Phe330, Leu326, Tyr322, Leu340, Lys342, Leu343, Val64, Ala61, Ile60
fulvestrant	−11.7	0	Tyr322, Pro312, Leu326, Ala61, Val64, Ile60, Leu340, Leu343, Lys342
dinaciclib	−10.2	1	Val313, Ile60, Ala61, Val64, Leu326, Leu340, Thr340
abemaciclib	−10	0	Arg323, Leu326, Ser316, Tyr322, Leu340, Leu343, Lys342, Val64, Ala61, Ile60
gemcitabine	−10.9	1	Val313, Ile57, Ala61, Ile60, Val64, Leu326, Leu340, Leu343, Phe330
voruciclib	−10.2	0	Val64, Ala61, Ile57, Ile60, Val313, Leu326, Leu340, Thr341, Leu343
fluorouracil	−11.6	0	Pro312, Tyr322, Leu326, Leu340, Leu343, Lys342, Ala61, Val64, Ile60
olaparib	−12.4	2	Val313, Ala61, Ile60, Ile57, Arg323, Leu326, Leu340, Lys342
ixabepilone	−11.7	2	Arg323, Ser316, Val313, Ala61, Val64, Ile60, Leu326, Leu340, Lys342, Leu343
alvocidib	−12.1	0	Val313, Ser316, Tyr322, Leu326, Leu340, Leu343, Lys342, Val64, Ala61, Ile60, Ile57
methotrexate	−9.7	2	Ser316, Pro312, Tyr322, Leu326, Val65, Ala61, Val64, Ile60, Phe330, Leu340, Leu343, Thr341, Lys342
ribociclib	−12.4	0	Ser198, Arg197, Cys196, Tyr195, Trp110, Asn108, Leu104, Lys40, Ser90, Phe94, Asn36, Asp30, Val28
exemestane	−13.6	0	Val313, Pro312, Ser316, Tyr322, Val65, Ala61, Ile57, Val64, Ile60, Leu326, Leu340, Leu343, Lys34
tamoxifen	−9.8	0	Ile66, Leu84, Ile70, Ile88, Leu91, Trp92
idarubicin	−10.1	1	Ser316, Tyr322, Leu326, Leu340, Leu343, Lys342, Ile57, Ile60, Ala61, Val64
palbociclib	−11.2	0	Val313, Ala61, Ile57, Ile60, Val64, Leu343, Leu326
toremifene	−9.9	0	Val313, Ile57, Ile60, Val64, Leu326, Ser350, Leu340, Leu343
roniciclib	−11	2	Met33, Pro35, Asn36, Asp30, Val28, Asn39, Pro38, Ile27, Leu43, Lys40, His107
capecitabine	−9.5	0	Ser316, Tyr322, Leu326, Ala61, Val64, Ile60, Ile57, Leu340, Leu343, Lys342
sulfanilamide	−10.6	1	Cys165, Arg162, Ile166, Leu84, Ile70, Trp92
zoledronic acid hydrate	−9.8	2	Val313, Ile60, Ala61, Ile57, Leu326, Leu340, Lys342, Thr341
pamidronic acid	−9.9	1	Val313, Ser316, Tyr322, Pro312, Leu326, Val64, Ala61, Ile60, Leu340, Lys34, Leu343
topotecan*	−10.1	0	Lys342, Leu343, Leu340, Phe330, Leu326, Ser316, Val313, Ala61, Ile57, Val64, Ile60
tucatinib*	−11	0	Lys342, Leu343, Leu340, Tyr322, Leu326, Val64, Ala61, Ile60, Ile67
pemetrexed*	−11.5	0	Lys342, Thr341, Leu343, Leu340, Phe330, TTyr322, Val64, Ser316, Ala61, Ile60
thiotepa*	−9.9	0	Lys342, Leu343, Leu340, Tyr322, Leu326, Ser316, Val313, Val64, Ile60

**Figure 3:**
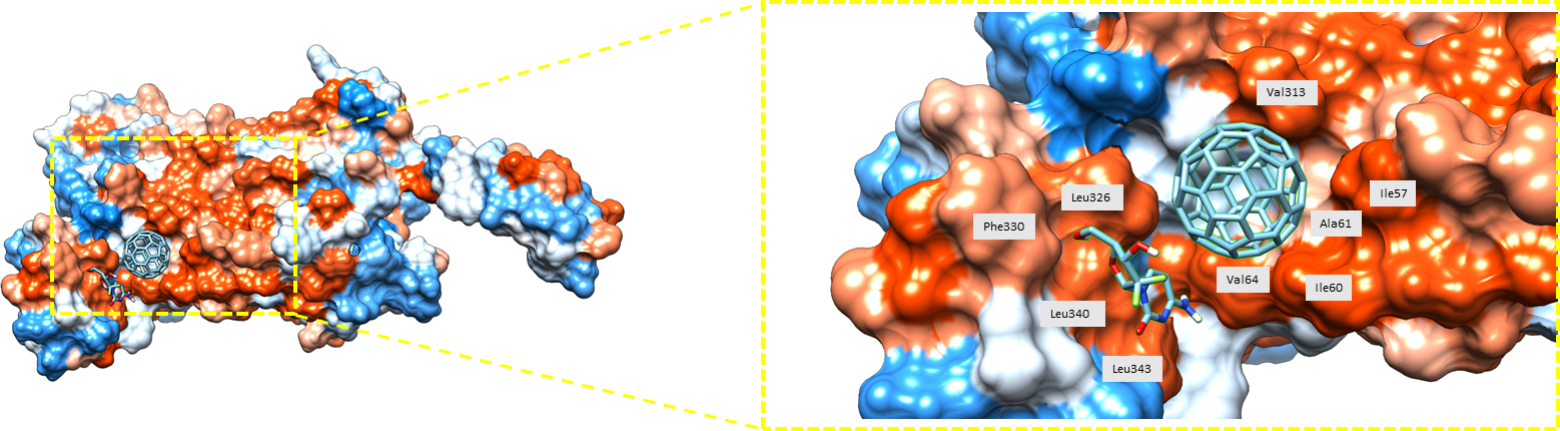
Binding site of gemcitabine–C_60_ and protein CXCR7. Blue color indicates the most hydrophilic sites and orange-red the most hydrophobic ones. Interacting residues are labeled as well.

The protein–ligand docking score between drug–C_60_ and CXCR7 varied from −13.6 to −9.2 kcal/mol. These values are consistently higher than those computed for the isolated drugs. In this case, examestane had the highest docking score, whereas epirubicine had the lowest docking score. The number of hydrogen bonds was between 0 and 2. Considering the results of the interacting residues, two things can be highlighted. First, drugs modified with fullerene C_60_ bind outside the protein; second, these binding sites are similar for the analyzed compounds (Figure 3). For example, the serine residue labeled as Ser316 is shared by 14 drugs, indicating that their binding zone is close to each other. Figure 3 shows the binding site between the protein and gemcitabine and the interacting residues.

The predictive QSAR/QSPR models were obtained as in the previous section. Table 6 shows the used quantum descriptors and the importance that each one has in the mathematical models for the drug–C_60_ complexes. The docking score was the predicted variable in all cases. The models for C_60_ were taken directly from the models of the isolated drugs. For example, model 1 for drug–C_60_ complexes considered variables with importance greater than zero from model 1 in the case of isolated drugs. The previous procedure was performed for all models. The most critical variables for the six models were LogP, p*K*_a_, and PSA. The least important variables in the six models were WS and Ac because they have less than 10% importance in the models.

**Table 6:** Input variables (IV) and output importance (OI) for six Extra Tree regressor models obtained from IBM Watson. Variables are annotated according to the notation introduced in Table 1. The best models, according to their MAPE values, are highlighted in bold.

Model 1		Model 2		Model 3		Model 4		Model 5		Model 6	
					
IV	OI (%)	IV	OI (%)	IV	OI (%)	IV	OI (%)	IV	OI (%)	IV	OI (%)

LogP	100	p*K*_a_	100	p*K*_a_	100	**p** ***K*** **_a_**	**100**	LogP	100	**MW**	**100**
PSA	97	LogP	55	PSA	69	**PSA**	**66**	Dn	58	***E*** **_LUMO_**	**96**
RBC	24	PSA	49	LogP	56	**LogS**	**32**	α	18	**ω**	**71**
*E*_HOMO_	23	*E*_LUMO_	29	*E*_LUMO_	24	***E*** **_LUMO_**	**25**	*E*_LUMO_	10	**NOR**	**34**
Dn	17	Dn	20	WS	8	**NOR**	**20**	NOR	6	**LogS**	**0**
ω	11	LogS	7	*E*_HOMO_	5	**WS**	**8**	LogS	1	—	—
α	8	*E*_HOMO_	1	ω	3	***E*** **_HOMO_**	**3**	WS	0	—	—
MW	4	Ac	0	α	3	**ω**	**1**	—	—	—	—
LogS	3	RBC	0	LogS	0	**MW**	**0**	—	—	—	—
NOR	1	MW	0	NOR	0	—	—	—	—	—	—
WS	1	NOR	0	—	—	—	—	—	—	—	—
Ac	0	WS	0	—	—	—	—	—	—	—	—

To give a quantitative reference about the performance of the predictive models, Supporting Information File 1, Table S4, shows the scores obtained for the validation set using both methods, the Extra Tree algorithm of IBM Watson and multiple linear regression. With these values, it is possible to appreciate the difference between the docking score obtained by molecular docking and the predictive models for the drug–C_60_ complexes. Table 7 shows the values obtained for the different evaluation metrics for each predictive model. The MSE ranges from 0.44 to 12.77 kcal^2^/mol^2^, and the MAPE varies from 4.97 to 31.5%. The MAE ranged from 0.5 to 3.26 kcal/mol, whereas the RMSE varied from 0.66 to 3.57 kcal/mol. The minimum error for all four metrics was obtained in MLR model 4, while the maximum error was obtained in MLR model 1. Considering the MAPE, the best model, with a value of 4.97%, to predict the docking score is MLR model 4. The explicit form of this model is:


[5] $$\begin{array}{c} {\text{MLR4}}_{\text{DRUG+C60}}=-10.253+0.179\times \text{p}K_{\text{a}}-0.003\times \text{PSA}\\ +0.292\times \text{LogS}+0.719\times E_{\text{LUMO}}\\ -0.071\times \text{NOR}-0.057\times \text{WS}\\ -1.020\times E_{\text{HOMO}}+0.006\times \omega . \end{array}$$

This linear model exhibited a higher performance than the non-exportable approaches provided by the AI. However, one needs to remember that the variables included in model 4 were selected by the initial AI screening. Thus, the selection of variables using AI offers a significant improvement for modeling using other mathematical methods. In addition, all metrics obtained in the case of MLR model 4 are better than those calculated in the case of the best MLR model of the isolated drugs and are comparable to those of the best AI model. Another significant MLR model is the model 2 with a MAPE of 7.58% and the following linear function:


[6] $$\begin{array}{c} {\text{MLR2}}_{\text{DRUG+C60}}=-12.884+0.141\times \text{p}K_{\text{a}}+0.139\times \text{LogP}\\ -0.10\times \text{PSA}+0.649\times E_{\text{LUMO}}\\ -0.261\times \text{Dn}+0.268\times \text{LogS}\\ -0.828\times E_{\text{HOMO}}. \end{array}$$

**Table 7:** Metrics obtained by using IA and MLR for drug–C_60_ complexes. The best models, according to their MAPE values, are highlighted in bold. The values were computed relative to the validation set.

Error	Model 1		Model 2		Model 3		Model 4		Model 5		Model 6	

	AI	MLR	AI	MLR	AI	MLR	AI	MLR	AI	MLR	AI	MLR

MSE (kcal^2^/mol^2^)	1.62	12.77	3.9	0.86	3.61	3.75	2.38	**0.44**	1.96	2.22	**0.93**	1.77
MAPE (%)	10.99	31.5	18.62	7.58	17.3	17.36	12.96	**4.97**	12.6	12.8	**7.53**	11.94
MAE (kcal/mol)	1.17	3.26	1.97	0.77	1.8	1.8	1.38	**0.5**	1.36	1.34	**0.82**	1.24
RMSE (kcal/mol)	1.27	3.57	1.97	0.92	1.9	1.94	1.54	**0.66**	1.4	1.49	**0.96**	1.33

The best AI model is model 6 with a MAPE of about 7.53% and the functional form AI6_DRUG+C60_ = *E*(MW, *E*_LUMO_, ω, NOR); all variables can be evaluated by theoretical approaches without the necessity of experimental results. The other significant model from AI, model 1, yielded a higher MAPE value of about 10.99%. In this case, the functional form is AI1_DRUG+C60_ = *E*(LogP, PSA, RBC, *E*_HOMO_, Dn, ω, α, MW, LogS, NOR, WS). Despite the large number of variables, including theoretical and experimental ones, the error is larger than those of the previously discussed models.

### Drugs modified with C_60_–COOH

To elucidate the effect of a fullerene derivative, the carboxyfullerene C_60_–COOH was chosen. A dataset with 19 drugs for the predictive model and four drugs as the validation set was built. The resultant dataset is shown in Supporting Information File 1, Table S5. As in the previous systems, the dataset was reduced to systems with less than 100 atoms. Because of this, ranges of the descriptors and their contributions to the predictive models were modified in the dataset. The molecular weights were increased to values ranging from 915.8022 to 1292.425 g/mol. As in the previous case, WS, LogS, p*K*_a_, and LogP are the same as those obtained for the isolated drugs. The hydrogen acceptor count varied between 3 and 13, whereas the hydrogen donor count varied from 1 to 7. Since the carboxylic group is polar, polar surface area values, ranging from 49.77 to 243.22 Å^2^, were modified. Also, after the introduction of the polar group, the RBC varied from 1 to 10.

The fullerene derivative C_60_–COOH was expected to modify the electronic structure of the composed systems. In consequence, the energy of the HOMO of the complexes was recomputed for the globally optimized systems at the DBTB3 level with solvation effects; the results ranged from −3.504 to −5.164 eV. Similarly, the energy of the LUMO varied from −3.48 to −4.437 eV. The electrophilicity computed by Koopman’s theorem had variations between 21.675 and 508.086 eV. Molecular docking was performed with the CXCR7 protein to obtain the docking score, number of established hydrogen bonds, and protein residues interacting with the complex at a distance of 3 Å. The results obtained with Autodock Vina for the 19 complexes and the validation set are shown in Table 8.

**Table 8:** Docking score, number of H-bonds established after docking, and interacting residues of CXCR7 with drug–fullerene C_60_−COOH complexes at 3 Å distance.

Ligand	Score (kcal/mol)	H-bonds	Interacting residues

dinaciclib	−9.7	0	Lys342, Leu340, Phe330, Leu343, Leu326, Tyr322, Val64, Ile60, Ala61
gemcitabine	−10.6	0	Phe294, Asn36, Asp30, Leu104, Arg197. Trp100, Tyr195, Ser190, Asn108, Lys40, Leu43
voruciclib	−10.3	0	Lys342, Leu343, Leu340, Val64, Leu326, Ser316, Val313, Ala61, Ile60, Ile57, Val56
fluorouracil	−11.4	1	Lys342, Leu343, Leu340, Thr341, Phe330, Leu326, Tyr322, Ser316, Val64, Ile60
olaparib	−11.6	0	Lys342, Leu343, Leu340, Phe330, Val64, Leu326, Tyr322, Arg323, Ser316, Ile60, Ala61, Val313
ixabepilone	−11.7	0	Ser190, Glu193, His107, Lys40, Arg197, Ile205, Pro38, Val28, Ile27
alvocidib	−10.9	1	Lys342, Leu343, Leu340, Val64, Leu326, Tyr322, Ala61, Ile60, Ile57
methotrexate	−10.9	2	Asn108, Lys40, Tyr195, Ser190, Arg197, Ser198, Tyr200, Leu43, Gln301, Tyr268, His298, Phe294, Asn39, Pro38, Asn36, Met37, His291, Asp30, Val28
ribociclib	−11.2	0	Leu104, Asn36, Phe294, Met33, Val32, Arg197, Ser190, Trp110, Tyr195, Asp30, Ile205, Lys206
exemestane	−11.6	1	Lys342, Leu343, Leu340, Leu326, Phe330, Ala61, Ile60, Ile57, Val313
tamoxifen	−9.1	0	Leu340, Leu326, Phe330, Ser316, Ile60, Val313, Ala61, Ile60, Ile57, Val56
idarubicin	−10.6	0	Leu343, Leu340, Lys342, Val64, Tyr322, Pro312, Ser316, Val313, Phe330, Ile60, Ile57
palbociclib	−11.4	0	Ile166, Arg162, Cys165, Cys81, Asn85, Ile88, Trp92, Ile70, Val66
toremifene	−9	0	Leu343, Lys342, Ser350, Leu340, Val64, Leu326, Ala61, Ser316, Ile60, Ile57
roniciclib	−9.2	0	Lys342, Leu343, Leu340, Leu326, Arg323, Val64, Ala61, Ile60, Ile57
capecitabine	−8.8	0	Thr341, Leu343, Leu340, Val64, Phe330, Leu326, Ala61, Ile60, Ile57, Val313
sulfanilamide	−10.7	1	Lys342, Leu343, Thr341, Leu340, Leu326, Tyr322, Ser316, Pro312, Val64, Ala61, Ile60
zoledronic acid hydrate	−10	0	Lys342, Leu343, Val64, Val65, Leu326, Tyr322, Ser316, Val313, Pro312, Ala61, Ile57
pamidronic acid	−10.6	3	Leu122, Ile126, Ile123, Trp169, Trp92, Ile166, Asn85, Cys165
topotecan*	−12.6	2	Thr341, Phe330, Leu340, Leu343, Leu326, Val64, Tyr322, Ser316, Val65, Pro312, Val313, Ala61, Ile60, Ile57
tucatinib*	−10.9	1	Leu343, Lys342, Leu340, Thr341, Val64, Tyr322, Leu326, Ser316, Val313, Ile60, Ala61, Val56, Ile57
pemetrexed*	−9.9	1	Ile166, Arg162, Trp92, Ile88, Leu84, Asn85, Thr75, Cys81
thiotepa*	−9.4	0	Lys342, Leu343, Leu340, Thr341, Val64, Leu326, Phe330, Ile60

The resultant docking score between drug–C_60_–COOH and CXCR7 ranged from −11.7 to −8.8 kcal/mol. As in the previous case, the water-soluble fullerene increased the docking score compared with the isolated drugs (Table 2). Modified ixabepilone had the highest docking score, and the capecitabine had the lowest. The numbers of hydrogen bonds were obtained in a narrow range from 0 to 3.

**Figure 4:**
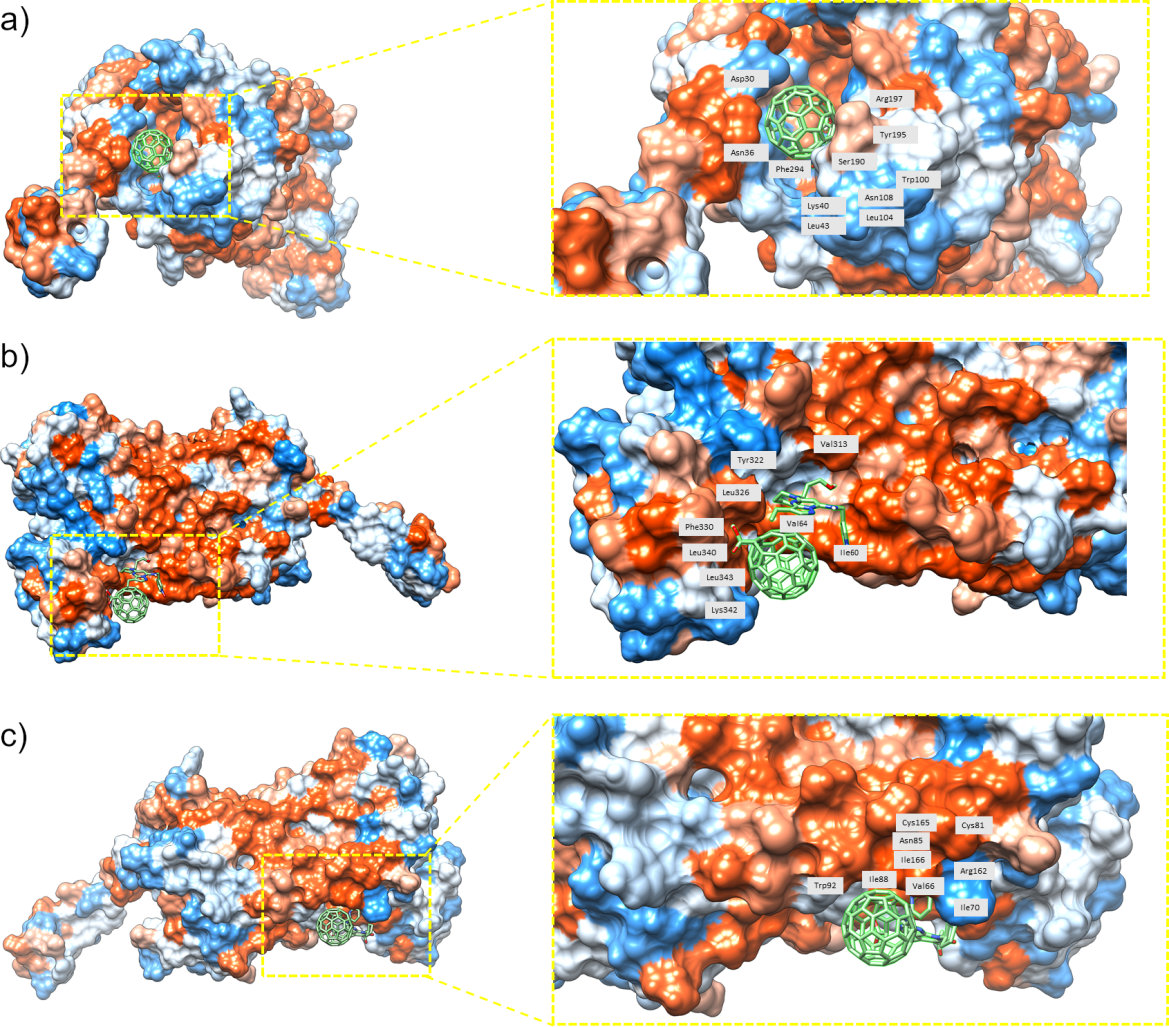
Binding sites for (a) gemcitabine, (b) dinaciclib, and (c) palbociclib with C_60_–COOH and protein CXCR7. Blue color indicates the most hydrophilic sites and orange-red the most hydrophobic ones. Interacting residues are labeled as well.

Considering the residues shown in Table 8 for the drug–fullerene complex with the protein, there are three possible binding sites. The first one is located inside the protein, near the pocket determined for isolated drugs. This binding site is near phenylalanine Phe294 and arginine Arg197 (Figure 4a) as prominent residues. The second possible binding site is outside the protein, near where drug–C_60_ binds; common residues are serine Ser316 and valine Val313 (Figure 4b). The third binding site is also at the outside of the protein and characterized by isoleucine Ile166 and arginine Arg162 (Figure 4c).

Table 9 shows the quantitative descriptors used and the importance that each one has in the predictive models for drug–C_60_–COOH. The docking score was the predicted variable in all the cases. In addition, the variables used to obtain the models in the case of drug–C_60_ were initially considered. The variables to model the interaction with C_60_–COOH were taken directly from those of drug–C_60_. For example, AI model 1 with C_60_–COOH used the variables that had an importance greater than zero from AI model 1 of drug–C_60_. The most important variables in the six models were LogP, RBC, and Dn. The least important variable in the six models was WS because had less than 10% importance in all models.

**Table 9:** Input variables (IV) and output importance (OI) obtained for six Extra Tree regressor models obtained from IBM Watson. Variables are annotated according to the notation introduced in Table 1. The best models, according to their MAPE values, are highlighted in bold.

Model 1		Model 2		Model 3		Model 4		Model 5		Model 6	

IV	OI (%)	IV	OI (%)	IV	OI (%)	IV	OI (%)	IV	OI (%)	IV	OI (%)

**RBC**	**100**	p*K*_a_	100	PSA	100	**Ω**	**100**	LogP	100	MW	100
**LogP**	**34**	LogP	96	LogP	77	**p** ***K*** **_a_**	**93**	LogS	90	NOR	96
**Dn**	**20**	Dn	69	p*K*_a_	72	**PSA**	**70**	α	83	*E*_LUMO_	38
**WS**	**10**	LogS	64	Α	42	***E*** **_LUMO_**	**31**	NOR	61	ω	0
**LogS**	**6**	*E*_HOMO_	30	*E*_HOMO_	32	**NOR**	**5**	Dn	52	—	—
**NOR**	**5**	PSA	12	*E*_LUMO_	26	***E*** **_HOMO_**	**2**	*E*_LUMO_	0	—	—
**ω**	**2**	*E*_LUMO_	0	WS	3	**LogS**	**1**	—	—	—	—
**MW**	**1**	—	—	ω	0	**WS**	**0**	—	—	—	—
***E*** **_HOMO_**	**0**	—	—	—	—	—	—	—	—	—	—
**PSA**	**0**	—	—	—	—	—	—	—	—	—	—
**α**	**0**	—	—	—	—	—	—	—	—	—	—

Supporting Information File 1, Table S6 shows the docking scores obtained for the validation set using both methods, that is, the Extra Tree regressors implemented in IBM Watson and the MLR. With these values, it is possible to compare the difference in the docking score obtained by molecular docking and the prediction of the mathematical models for drug–C_60_–COOH and the protein CXCR7. Table 10 shows the values obtained for the different types of errors in each of the models. The MSE ranged from 0.77 to 4.73 kcal^2^/mol^2^, whereas the MAPE varied from 6.70 to 16.22%. Also, the MAE was obtained ranging from 0.69 to 6.52 kcal/mol. Finally, the RMSE varied from 0.88 to 2.18 kcal/mol. Considering the MAPE, the best model, with a value of 6.7%, to predict the docking score is MLR model 1. Once again, the synergistic effect of using AI with a mathematical tool such as MLR is observed. The benefits are the predictive model’s clarity, whereas AI was useful in determining the most important descriptors to be included in the QSAR/QSPR model. The explicit form of this model is:


[7] $$\begin{array}{c} {\text{MLR1}}_{\text{DRUG+C60-COOH}}=-13.597+0.047\times \text{p}K_{\text{a}}+0.33\times \text{LogP}\\ +0.18\times \text{Dn}+0.284\times \text{LogS}\\ -0.696\times E_{\text{HOMO}}-0.007\times \text{PSA.} \end{array}$$

The other significant model is MLR model 5 with a MAPE of 10.17%. In this model, experimental and theoretical descriptors were mixed. The following is the explicit form of MLR model 5:


[8] $$\begin{array}{c} {\text{MLR5}}_{\text{DRUG+C60-COOH}}=2.480+0.552\times \text{LogP}\\ +0.21\times \text{LogS}+0.002\times \alpha \\ -0.402\times \text{NOR}+0.186\times \text{Dn.} \end{array}$$

Considering the MAPE, the best AI model is model 4 with a value of 8.18% and the functional form AI4_DRUG+C60-COOH_ = *f* (*E*_HOMO_, ω, p*K*_a_, PSA, *E*_LUMO_, LogS, NOR). The other significant model obtained from AI is model 5, with a MAPE value of 8.69%; the functional form of this model is AI5_DRUG+C60-COOH_ = *f* (LogP, α, LogS, NOR, Dn). Thus, although the Extra Trees algorithm was competitive in the case of drugs modified with a carboxyfullerene, this approach was surpassed by the MLR with the AI choosing the most important variables.

**Table 10:** Metrics obtained by the use of AI and MLR in the case of drug–C_60_−COOH. The best models, according to their MAPE values, are highlighted in bold. Values were computed relative to the validation set.

Error	Model 1		Model 2		Model 3		Model 4		Model 5		Model 6	

	AI	MLR	AI	MLR	AI	MLR	AI	MLR	AI	MLR	AI	MLR

MSE (kcal^2^/mol^2^)	0.97	**0.77**	1.67	1.77	1.80	4.73	**1.05**	1.53	1.41	2.08	1.46	1.80
MAPE (%)	8.75	**6.70**	10.51	12.03	10.71	16.22	**8.18**	10.26	8.69	10.17	10.86	11.39
MAE (kcal/mol)	0.93	**0.69**	1.13	1.29	1.17	6.52	**0.86**	1.10	0.93	0.99	1.16	1.18
RMSE (kcal/mol)	0.98	**0.88**	1.29	1.33	1.34	2.18	**1.02**	1.24	1.19	1.44	1.21	1.34

### Doxorubicin and gemcitabine with a water-soluble fullerene

**Figure 5:**
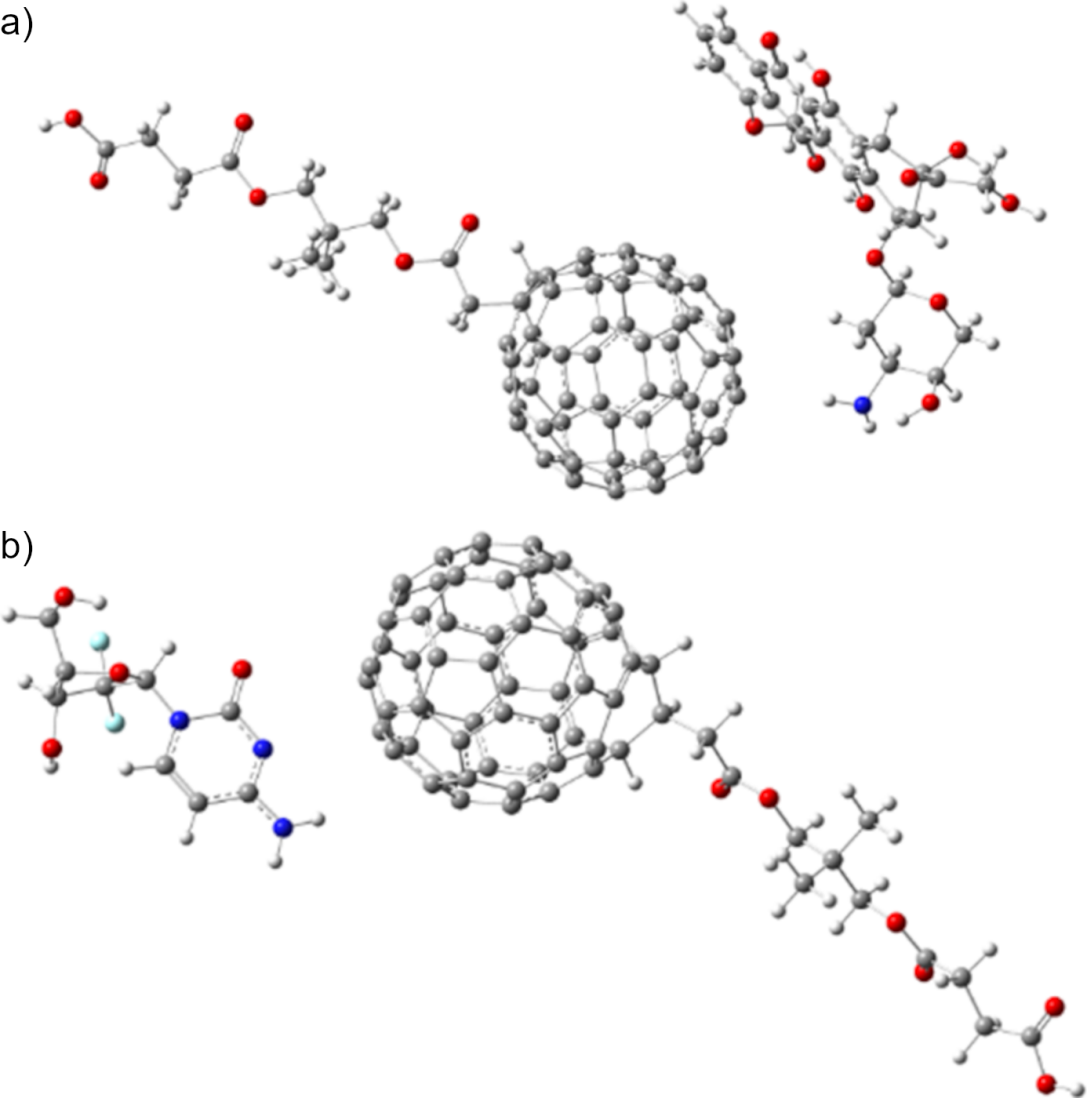
Ground state structures of (a) doxorubicin and (b) gemcitabine interacting with a water-soluble fullerene.

Finally, doxorubicin and gemcitabine were selected to compare the DFTB3 approach with the regular DFT method. In addition, their interactions with a water-soluble fullerene derivative were studied as well. Both anticancer agents are presented in Figure 5 interacting with a water-soluble fullerene [36–37]. Doxorubicin, an antibiotic that belongs to the family of tetracycline pharmaceutical agents, has gained popularity among chemotherapy agents and was recently modified with fullerene C_60_ [72–75]. Its anticarcinogenic activity comes from its ability to intercalate into DNA, inducing damage of the DNA strands and inhibiting its replication. Also, doxorubicin contributes to stopping the action of the enzyme topoisomerase II, leading to apoptosis of living tissues [71]; therefore, it is important to study its intrinsic chemical reactivity.

At the B3PW91/6-31G level of DFT, it is possible to appreciate that the periphery of the doxorubicin molecule is saturated by organic substituents. Its oxy, carbonyl, and carboxy terminal groups are active sites to interact with DNA or amino acids. The molecular orbital scheme of this molecule is shown in Supporting Information File 1, Figure S2, together with its molecular electrostatic potential (ESP). In the case of doxorubicin, from frontier molecular orbitals theory, HOMO and LUMO were found on the tetracycline moiety. However, the HOMO is confined to the quinoid ring, whereas the LUMO is completely delocalized (Supporting Information File 1, Figure S2). Oxygen atoms are the regions with the most negative ESP (red color in Supporting Information File 1, Figure S2). In contrast, a high electrostatic potential (blue color in Supporting Information File 1, Figure S2) is found on the lateral substituted cycle. Both central rings, the aromatic one and the quinoid one, are the main regions for the reactivity, including all the substituent oxygen atoms. The frontier molecular orbitals are similar, and it is expected that electronic transit can occur in this region accepting and donating negative charges. The ESP map reinforces this suggestion showing negative density sites as well as a positive center, which can receive electrons. The energy of the HOMO was computed as −5.978 eV and that of the LUMO as −4.221 eV at the DFTB3 level. In comparison, the B3PW91 method yielded −6.116 and −3.242 eV, respectively. Thus, to consider the models obtained here, it is recommended to use DFTB3 to compute the electronic and energetic properties instead of DFT calculations.

Gemcitabine includes an active pyrimidinone fragment as a very reactive zone. The frontier molecular orbitals and the ESP map are shown Supporting Information File 1, Figure S2. Again, there is a strong polarization, which can induce a route for reaction. The ring nitrogen atom in alpha position concerning the carbonyl group is the more nucleophilic center, whereas there are two positive-density regions near the carbonyl group and in the C–C bond next to the amine-substituted carbon atom.

Both pharmaceutical agents are susceptible to interaction with fullerenes to form a force dispersion complex as it has been previously suggested. However, these complexes should be water-soluble to be delivered to their host. Considering all these factors, a water-soluble species was used to form such complexes, the structure of which [76] is shown in Figure 5. In both cases, a strong hydrogen bond is present; the distances are 1.97 Å for the doxorubicin complex and 2.25 Å for the gemcitabine complex. Furthermore, the energy of these interactions was calculated taking advantage of the Grimme module; they are 23.7 kcal/mol for doxorubicin and 18.9 kcal/mol for gemcitabine.

## Conclusion

A QSAR/QSPR study of drugs commonly used for breast cancer chemotherapy modified with fullerene derivatives as drug nanocarriers was carried out. The CXCR7 protein was selected as a target for molecular docking calculations; the drugs were studied in the isolated form and modified with C_60_ fullerene and with the water-soluble C_60_–COOH fullerene derivative. An initial dataset was built by analyzing more than 30 drugs. The models to predict the docking score were obtained concerning Pearson’s HSAB concept and common QSAR/QSPR descriptors. The energetic descriptors were computed quantum chemically by using density functional-based tight binding at the DFTB3 level. The highest docking score in the case of isolated drugs was −10.1 kcal/mol for olaparib. In contrast, in the case of the drugs modified with pristine C_60_ fullerene, it was −13.6 kcal/mol for exemestane. In the case of the drugs modified with the water-soluble fullerene derivative C_60_–COOH, the maximum docking score was −11.7 kcal/mol for ixabepilone. Hence, the complexes are supposed to dock with stronger interactions with the CXCR7 protein than the isolated drugs. Also, characteristic binding sites were determined. The pocket of the isolated drugs was found within the protein, sharing residues including Trp100, Leu297, and Ser198. In the case of the drugs with fullerene C_60_, the binding site was outside the protein with the complex pointing away from the pocket. The interacting residues included Arg323, Ser316, and Lys342. In the case of the drugs with C_60_–COOH fullerene, there were three possible binding sites. The first two are the same as those in the previous cases The third binding site was found outside the protein, near the residues Ile166, Arg192, and Cys165. The docking score for the drug–fullerene complex is higher than that of the isolated drugs. QSAR/QSPR predictive models for the docking score were obtained from MLR and from IBM Watson artificial intelligence, yielding models with a MAPE of lower than 12% in all three cases. Although MLR exhibits the best evaluation metrics in the case of drug–C_60_ and drug–C_60_–COOH complexes, an improvement is obtained based on the variables detected by the AI as the most important ones.

## Supporting Information

File 1 Additional tables and figures.

## Data Availability

The data that supports the findings of this study is available from the corresponding author upon reasonable request.
