# Supplementary material for: AI-assisted models to predict chemotherapy drugs modified with C60 fullerene derivatives
Source: Beilstein J Nanotechnol. 2024 Sep 19;15:1170–88. doi: 10.3762/bjnano.15.95 (PMC11420546; doi:10.3762/bjnano.15.95)
Supplement: File 1 — Additional tables and figures. [file Beilstein_J_Nanotechnol-15-1170-s001.pdf]

BEILSTEIN JOURNAL OF NANOTECHNOLOGY

Supporting Information

for

AI-assisted models to predict chemotherapy drugs modified with C_{60} fullerene derivatives

Jonathan-Siu-Loong Robles-Hernández, Dora Iliana Medina, Katerin Aguirre-Hurtado, Marlene Bosquez, Roberto Salcedo and Alan Miralrio

Beilstein J. Nanotechnol. **2024,** *15,* 1170–1188. doi:10.3762/bjnano.15.95

Additional tables and figures

License and Terms: This is a supporting information file under the terms of the Creative Commons Attribution License (https://creativecommons.org/licenses/by/4.0). Please note that the reuse, redistribution and reproduction in particular requires that the author(s) and source are credited and that individual graphics may be subject to special legal provisions.
The license is subject to the *Beilstein Journal of Nanotechnology* terms and conditions: (https://www.beilstein-journals.org/bjnano/terms)

Table S1: Dataset with the 14 quantitative descriptors used to analyze the case of isolated drugs.

Drug name	MW [g/mol]	WS [mg/ml]	Log P	Log S	pKa	Ac	Dn	PSA [Å^2]	RBC	α [Å^3]	NOR	♦ E_{HOMO} [eV]	♦ E_{LUMO} [eV]	♦ ω [eV]
Doxorubicin	543.52	1.1800	1.41	-2.7	10.030	12	6	206.07	5	54.62	5	-5.978	-4.221	14.801
Neratinib maleate	557.04	0.0067	4.47	-4.9	8.810	8	2	112.40	13	58.21	4	-5.239	-2.744	6.386
Epirubicin	543.50	0.0930	1.30	-2.7	10.030	12	6	206.07	5	54.62	5	-5.971	-4.202	14.625
Lapatinib Ditosylate	915.40	0.0223	4.64	-4.4	6.910	7	2	106.35	13	61.19	7	-5.504	-5.341	180.390
Fulvestrant	606.78	0.0067	6.54	-5.0	-0.880	3	2	57.53	15	65.88	4	-5.545	-1.276	2.725
Dinaciclib	396.50	0.0524	2.40	-3.9	4.630	6	2	92.63	7	44.18	4	-4.765	-2.430	5.543
Abemaciclib	506.61	0.0159	4.25	-4.5	7.940	7	1	75.00	7	54.75	5	-4.828	-2.852	7.462
Gemcitabine	263.20	22.3000	0.14	-1.1	3.650	6	3	108.38	2	21.62	2	-6.464	-2.422	4.884
Voruciclib	469.84	0.0363	3.91	-4.1	7.790	6	3	90.23	4	42.90	4	-5.146	-3.443	10.830
Fluorouracil	130.08	5.8600	-0.58	-1.4	-8.000	2	2	58.20	0	9.46	1	-6.682	-2.871	5.987
Letrozole	285.30	0.0799	1.86	-3.6	1.890	4	0	78.29	3	29.59	3	-6.534	-2.726	5.629
Olaparib	434.46	0.0601	2.72	-3.9	-0.900	4	1	82.08	4	43.81	5	-6.099	-2.691	5.668
Paclitaxel	853.91	0.0056	3.20	-5.2	-1.200	10	4	221.29	14	87.15	7	-6.108	-2.811	6.032
Seliciclib	354.45	0.1630	3.11	-3.3	5.200	6	3	87.89	8	40.43	3	-5.097	-1.456	2.948
Ixabepilone	506.70	0.0035	3.28	-5.2	2.730	6	3	112.05	2	56.86	3	-5.671	-2.120	4.273
Anastrozole	293.37	0.0066	2.31	-3.6	2.000	4	0	78.29	4	31.97	2	-6.408	-1.856	3.751
Pentostatin	268.27	10.7000	-2.00	-1.4	8.330	7	4	112.13	2	26.34	3	-5.056	-0.889	2.120
Alvocidib	401.84	0.0940	2.81	-3.6	7.700	6	3	90.23	2	40.85	4	-5.355	-3.068	7.755
Methotrexate	454.44	0.0819	-0.05	-3.7	14.550	12	6	205.92	9	45.43	3	-5.380	-3.123	8.009
Milciclib	460.59	0.0994	3.15	-3.7	7.960	7	2	91.21	4	52.87	5	-4.561	-2.345	5.381
Ribociclib	434.55	0.2310	2.50	-3.3	8.870	7	2	91.21	5	49.13	5	-4.613	-2.249	4.980
Exemestane	296.40	0.0068	2.67	-4.6	-5.000	2	0	34.14	0	33.72	4	-5.973	-3.134	7.303
Tamoxifen	371.51	0.0010	5.93	-5.6	8.760	2	0	12.47	8	44.19	3	-5.188	-1.745	3.490
Idarubicin	497.49	0.7720	1.69	-2.8	10.040	10	5	176.61	3	50.84	5	-6.070	-4.275	14.905
Palbociclib	447.53	0.0174	2.12	-4.4	8.860	8	2	103.35	5	49.69	5	-4.752	-3.019	8.712
Toremifene	405.96	0.0004	5.65	-6.0	8.760	2	0	12.47	9	46.49	3	-5.198	-1.808	3.620
Vinblastine	810.97	0.0169	4.22	-4.7	8.860	9	3	154.10	10	87.46	9	-4.392	-1.488	2.976
Pirarubicin	627.64	0.3010	2.06	-3.3	9.090	13	5	204.30	7	64.85	6	-5.901	-4.185	14.820
Roniciclib	430.45	0.0496	3.24	-3.9	2.500	7	3	108.19	8	39.89	3	-6.479	-3.176	7.056
Capecitabine	359.35	0.2480	1.17	-3.2	0.073	6	3	120.69	7	35.94	2	-6.417	-3.250	7.377
Sulfanilamide	172.21	10.4000	-0.16	-1.2	2.270	3	2	86.18	1	16.25	1	-6.380	-4.437	15.055
Zoledronic acid hydrate	272.09	3.2700	-0.93	-1.9	6.670	8	5	153.11	4	20.10	1	-6.060	-1.889	3.787
Pamidronic acid	235.07	15.8000	-1.40	-1.2	9.860	8	6	161.31	4	17.34	0	-7.400	-1.487	3.339
Docetaxel*	807.88	0.0127	2.92	-4.8	-3.000	10	5	224.45	13	82.15	6	-5.241	-2.689	6.160
Topotecam*	421.45	0.8610	-0.33	-2.7	9.750	6	2	103.20	3	44.86	5	-5.320	-3.317	9.311
Tucatimib*	480.53	0.0040	5.25	-5.1	4.570	8	2	110.85	5	50.76	6	-5.305	-2.632	5.892
Aldoxorubicin*	750.76	0.1510	0.71	-3.7	9.390	15	7	267.84	12	75.89	6	-5.797	-4.067	14.060
Vinorelbine*	778.95	0.0122	4.65	-4.8	8.660	8	2	133.87	10	84.31	9	-4.861	1.581	3.163
Etoposide*	588.56	0.9780	1.16	-2.8	-3.700	12	3	160.83	5	57.95	7	-5.205	-1.423	2.904
Pemetrexed*	427.41	0.0455	1.20	-4.0	2.430	9	6	186.97	9	43.24	3	-6.086	-2.567	5.319
Everolimus*	958.24	0.0016	7.40	-5.8	-2.700	13	3	204.66	9	106.61	4	-5.408	-3.203	8.407
Thiotepa*	189.22	9.2700	-1.00	-1.3	-0.300	3	0	9.03	3	18.22	3	-6.297	0.711	1.113

Represent the drugs used in the validation set. ♦ Are the descriptors obtained by DFTB3 theory.

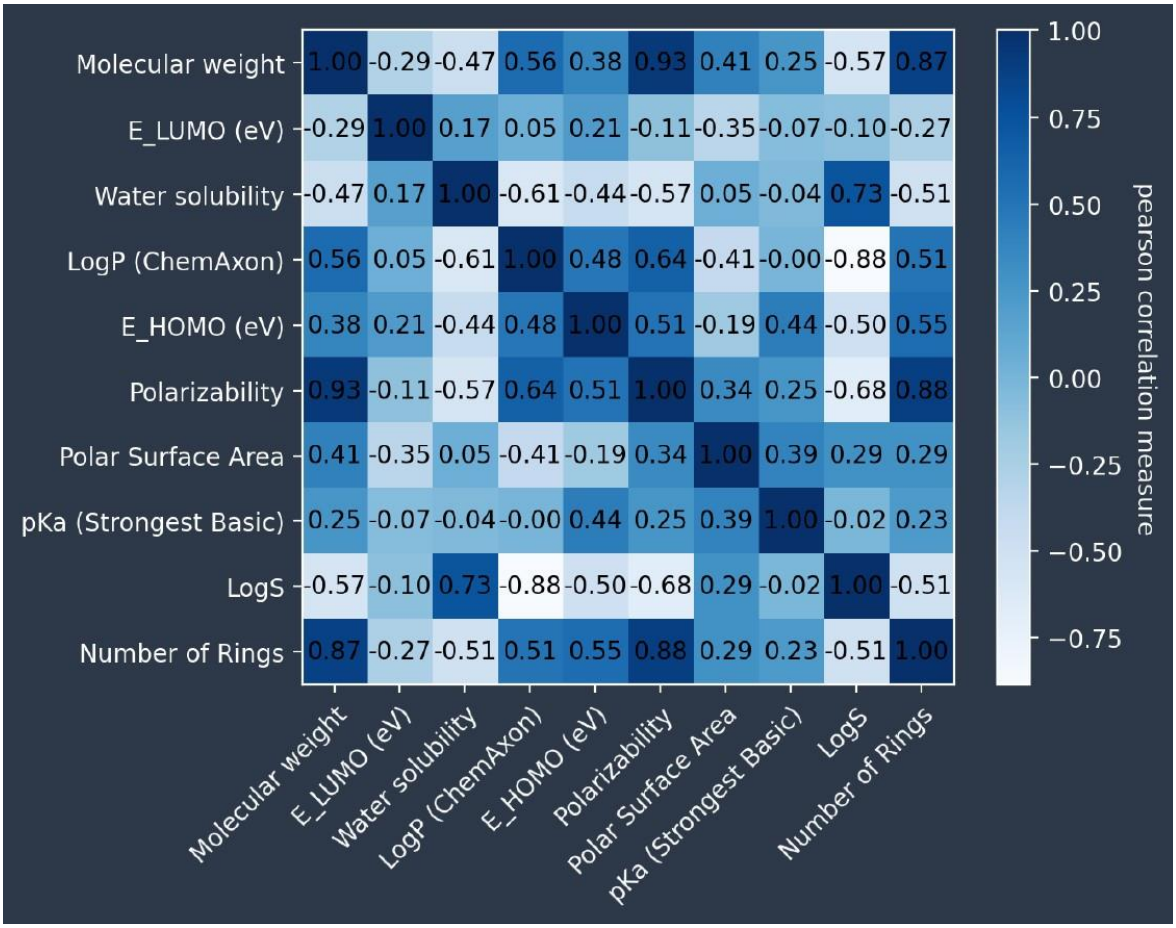

Figure S1: Correlation matrix between the ten most important descriptors.

Table S2: Docking scores were obtained from the validation set for drugs modeled using AI and MLR.

	Docking score (kcal/mol)	Model 1 (kcal/mol)		Model 2 (kcal/mol)		Model 3 (kcal/mol)		Model 4 (kcal/mol)		Model 5 (kcal/mol)		Model 6 (kcal/mol)	
**Drug name**	**-**	**AI**	**MLR**	**AI**	**MLR**	**AI**	**MLR**	**AI**	**MLR**	**AI**	**MLR**	**AI**	**MLR**
Docetaxel	-9.5	-9.7	-8.9	-9.6	-8.9	-9.6	-9.1	-9.6	-8.8	-9.5	-9.3	-9.6	-9.4
Topotecan	-8.2	-8.8	-7.8	-7.8	-7.8	-8.1	-8.0	-8.1	-8.8	-8.3	-8.5	-8.0	-8.5
Tucatimib	-10.1	-8.5	-10.5	-8.9	-10.4	-9.5	-10.4	-9.5	-9.6	-8.9	-9.4	-9.0	-9.8
Aldoxorubicin	-8.4	-9.2	-9.0	-8.8	-9.1	-8.1	-9.1	-8.1	-9.3	-9.1	-9.2	-9.0	-9.3
Vinorelbine	-9.1	-9.3	-10.6	-9.0	-10.4	-9.5	-10.7	-9.5	-10.7	-9.5	-10.5	-9.3	-10.7
Etoposide	-9.3	-9.0	-10.4	-8.8	-10.2	-9.5	-9.2	-9.5	-8.8	-8.7	-9.3	-8.8	-8.9
Pemetrexed	-9.1	-8.4	-7.3	-8.3	-7.4	-8.3	-8.1	-8.4	-7.7	-8.3	-7.8	-8.4	-7.8
Everolimus	-9.2	-8.7	-10.5	-8.5	-10.9	-8.2	-7.9	-7.7	-8.0	-8.7	-8.7	-8.4	-8.7
Thiotepa	-3.9	-5.8	-6.3	-5.5	-6.4	-4.6	-5.7	-4.6	-6.3	-5.9	-6.6	-5.8	-6.0

Table S3: Dataset with the quantitative descriptors used to analyze the case of drugs with fullerene C_{60}.

Drug name	▲ MW [g/mol]	WS [mg/ml]	Log P	Log S	pKa	Ac	Dn	PSA [Å²]	RBC	α [Å³]	▲ NOR	♦ E_{HOMO} [eV]	♦ E_{LUMO} [eV]	♦ ω [eV]
Doxorubicin	1320.61	1.1800	1.41	-2.7	10.030	12	6	206.07	5	54.62	37	-5.697	-4.117	15.240
Epirubicin	1320.59	0.0930	1.30	-2.7	10.030	12	6	206.07	5	54.62	37	-5.672	-4.185	16.335
Lapatinib Ditosylate	1692.49	0.0223	4.64	-4.4	6.910	7	2	106.35	13	61.19	39	-5.237	-3.896	15.550
Fulvestrant	1383.87	0.0067	6.54	-5.0	-0.880	3	2	57.53	15	65.88	36	-5.532	-3.917	13.821
Dinaciclib	1173.58	0.0524	2.40	-3.9	4.630	6	2	92.63	7	44.18	36	-4.736	-3.951	24.033
Abemaciclib	1283.69	0.0159	4.25	-4.5	7.940	7	1	75.00	7	54.75	37	-3.617	-3.571	280.801
Gemcitabine	1040.28	22.3000	0.14	-1.1	3.650	6	3	108.38	2	21.62	34	-5.693	-3.934	13.172
Voruciclib	1246.93	0.0363	3.91	-4.1	7.790	6	3	90.23	4	42.90	36	-4.867	-3.277	10.428
Fluorouracil	907.16	5.8600	-0.58	-1.4	-8.000	2	2	58.20	0	9.46	33	-5.701	-3.943	13.226
Olaparib	1211.55	0.0601	2.72	-3.9	-0.900	4	1	82.08	4	43.81	37	-5.673	-3.918	13.104
Ixabepilone	1283.79	0.0035	3.28	-5.2	2.730	6	3	112.05	2	56.86	35	-5.577	-3.956	14.016
Alvocidib	1178.93	0.0940	2.81	-3.6	7.700	6	3	90.23	2	40.85	36	-4.765	-3.182	9.974
Methotrexate	1231.53	0.0819	-0.05	-3.7	14.550	12	6	205.92	9	45.43	35	-5.335	-3.942	15.446
Ribociclib	1211.63	0.2310	2.50	-3.3	8.870	7	2	91.21	5	49.13	37	-3.458	-3.549	-134.885
Exemestane	1073.49	0.0068	2.67	-4.6	-5.000	2	0	34.14	0	33.72	36	-5.631	-5.388	124.916
Tamoxifen	1148.60	0.0010	5.93	-5.6	8.760	2	0	12.47	8	44.19	35	-4.771	-3.179	9.925
Idarubicin	1274.58	0.7720	1.69	-2.8	10.040	10	5	176.61	3	50.84	37	-5.486	-3.853	13.352
Palbociclib	1224.62	0.0174	2.12	-4.4	8.860	8	2	103.35	5	49.69	37	-4.473	-3.917	31.651
Toremifene	1183.05	0.0004	5.65	-6.0	8.760	2	0	12.47	9	46.49	35	-4.807	-3.210	10.061
Roniciclib	1207.54	0.0496	3.24	-3.9	2.500	7	3	108.19	8	39.89	35	-5.672	-5.002	42.513
Capecitabine	1136.44	0.2480	1.17	-3.2	0.073	6	3	120.69	7	35.94	34	-5.718	-3.966	13.382
Sulfanilamide	949.29	10.4000	-0.16	-1.2	2.270	3	2	86.18	1	16.25	33	-5.682	-4.445	20.727
Zoledronic acid hydrate	1049.18	3.2700	-0.93	-1.9	6.670	8	5	153.11	4	20.10	33	-5.374	-3.756	12.880
Pamidronic acid	1012.16	15.8000	-1.40	-1.2	9.860	8	6	161.31	4	17.34	32	-4.796	-3.204	10.050
Topotecam*	1142.11	0.8610	-0.33	-2.7	9.750	6	2	103.20	3	44.86	37	-3.775	-3.502	48.493
Tucatimib*	1201.19	0.0040	5.25	-5.1	4.570	8	2	110.85	5	50.76	38	-5.396	-3.921	14.713
Pemetexed*	1148.07	0.0455	1.20	-4.0	2.430	9	6	186.97	9	43.24	35	-5.343	-4.117	18.249
Thiotepa*	909.88	9.2700	-1.00	-1.3	-0.300	3	0	9.03	3	18.22	35	-4.828	-3.243	10.275

**Represent the drugs used in the validation set. ♦ Are the descriptors obtained by DFTB3 theory.▲ Show the variables modified by the fullerene C_{60}. The other descriptors are the same that for the isolated case.*

Table S4: Docking scores were obtained from the validation set for drugs with fullerene C60, using AI and MLR.

	Docking score (kcal/mol)	Model 1 (kcal/mol)		Model 2 (kcal/mol)		Model 3 (kcal/mol)		Model 4 (kcal/mol)		Model 5 (kcal/mol)		Model 6 (kcal/mol)	
**Drug name**	-	**AI**	**MLR**	**AI**	**MLR**	**AI**	**MLR**	**AI**	**MLR**	**AI**	**MLR**	**AI**	**MLR**
Topotecan	-10.1	-12.1	-10.91	-11.6	-13.34	-12.39	-11.95	-12.4	-10.66	-11.12	-11.37	-11.12	-11.49
Tucatinib	-11	-9.34	-10.36	-11.7	-9.11	-10	-9.83	-11.7	-11.17	-9.4	-10.67	-10.12	-11.99
Pemetrexed	-11.5	-9.5	-11.38	-9.7	-9.23	-10.1	-10.28	-9.7	-11.40	-9.7	-9.74	-10.12	-10.88
Thiotepa	-9.9	-12.1	-11.43	-10.6	-15.54	-12.4	-12.85	-10.6	-11.09	-10.9	-11.91	-9.9	-11.84

Table S5: Dataset with the quantitative descriptors used to analyze the case of drugs with fullerene C_{60}-COOH.

Drug name	▲MW [g/mol]	WS [mg/ml]	Log P	Log S	pKa	▲ Ac	▲ Dn	▲ PSA [Å²]	▲ RBC	α [Å³]	NOR	♦ E_{HOMO} [eV]	♦ E_{LUMO} [eV]	♦ ω [eV]
Dinaciclib	1182.22	0.0524	2.40	-3.9	4.630	7	3	129.93	8	44.18	36	-4.958	-4.063	22.731
Gemcitabine	1048.92	22.3000	0.14	-1.1	3.650	7	4	145.68	3	21.62	34	-5.164	-4.314	26.421
Voruciclib	1255.57	0.0363	3.91	-4.1	7.790	7	4	127.53	5	42.90	36	-4.903	-4.035	23.009
Fluorouracil	915.80	5.8600	-0.58	-1.4	-8.000	3	3	95.50	1	9.46	33	-5.140	-4.293	26.264
Olaparib	1220.19	0.0601	2.72	-3.9	-0.900	5	2	119.38	5	43.81	37	-5.056	-4.257	27.138
Ixabepilone	1292.43	0.0035	3.28	-5.2	2.730	7	4	149.35	3	56.86	35	-5.112	-4.254	25.560
Alvocidib	1187.57	0.0940	2.81	-3.6	7.700	7	4	127.53	3	40.85	36	-5.150	-4.294	26.048
Methotrexate	1240.16	0.0819	-0.05	-3.7	14.550	13	7	243.22	10	45.43	35	-5.094	-4.258	26.154
Ribociclib	1220.27	0.2310	2.50	-3.3	8.870	8	3	128.51	6	49.13	37	-3.504	-3.480	508.086
Exemestane	1082.13	0.0068	2.67	-4.6	-5.000	3	1	71.44	1	33.72	36	-5.121	-4.263	25.658
Tamoxifen	1157.24	0.0010	5.93	-5.6	8.760	3	1	49.77	9	44.19	35	-5.058	-4.210	25.323
Idarubicin	1283.22	0.7720	1.69	-2.8	10.040	11	6	213.91	4	50.84	37	-5.131	-4.283	26.127
Palbociclib	1233.26	0.0174	2.12	-4.4	8.860	9	3	140.65	6	49.69	37	-4.709	-4.027	27.976
Toremifene	1191.69	0.0004	5.65	-6.0	8.760	3	1	49.77	10	46.49	35	-4.908	-3.994	21.675
Roniciclib	1216.18	0.0496	3.24	-3.9	2.500	8	4	145.49	9	39.89	35	-5.130	-4.288	26.336
Capecitabine	1145.08	0.2480	1.17	-3.2	0.073	7	4	157.99	8	35.94	34	-5.093	-4.282	27.093
Sulfanilamide	957.93	10.4000	-0.16	-1.2	2.270	4	3	123.48	2	16.25	33	-5.151	-4.437	32.188
Zoledronic acid hydrate	1057.81	3.2700	-0.93	-1.9	6.670	9	6	190.41	5	20.10	33	-5.151	-4.301	26.277
Pamidronic acid	1020.79	15.8000	-1.40	-1.2	9.860	9	7	198.61	5	17.34	32	-5.162	-4.324	26.845
Topotecam*	1150.74	0.8610	-0.33	-2.7	9.750	7	3	140.50	4	44.86	37	-5.129	-4.281	26.105
Tucatimib*	1209.83	0.0040	5.25	-5.1	4.570	9	3	148.15	6	50.76	38	-5.129	-4.281	26.105
Pemetrexed*	1156.71	0.0455	1.20	-4.0	2.430	10	7	224.27	10	43.24	35	-5.148	-4.297	26.207
Thiotepa*	918.52	9.2700	-1.00	-1.3	-0.300	4	1	46.33	4	18.22	35	-5.107	-4.297	27.295

*Represent the drugs used in the validation set. ♦ Are the descriptors obtained by DFTB3 theory. ▲ Show thevariables modified by the fullerene C_{60}–COOH. The other descriptors are the same that for previous cases.

Table S6: Docking scores obtained from the validation set in the case of drugs with fullerene C_{60}–COOH, by the use of AI and MLR. The best models, according to their MAPEs, are highlighted.

	Docking score (kcal/mol)	Model 1 (kcal/mol)		Model 2 (kcal/mol)		Model 3 (kcal/mol)		Model 4 (kcal/mol)		Model 5 (kcal/mol)		Model 6 (kcal/mol)	
**Drug name**	**-**	**AI**	**MLR**	**AI**	**MLR**	**AI**	**MLR**	**AI**	**MLR**	**AI**	**MLR**	**AI**	**MLR**
Topotecan	-12.6	-11.39	-11.85	-10.96	-10.85	-10.58	-13.14	-11.39	-10.81	-10.87	-12.50	-11.43	-11.42
Tucatinib	-10.9	-11.39	-10.51	-9.85	-9.99	-9.98	-8.89	-10.89	-11.16	-10.88	-10.31	-9.32	-11.59
Pemetrexed	-9.9	-9.19	-9.99	-9.76	-10.90	-9.64	-10.05	-9.19	-10.88	-9.49	-10.38	-9.29	-10.51
Thiotepa	-9.4	-10.69	-10.94	-11.09	-10.89	-10.88	-13.22	-10.89	-10.77	-10.97	-12.18	-10.67	-11.63

Table S7: Cross-validation obtained for the MLR models in case of the isolated drugs.

**Error**	**Model 1**	**Model 2**	**Model 3**	**Model 4**	**Model 5**	**Model 6**
	**MLR**	**MLR**	**MLR**	**MLR**	**MLR**	**MLR**
MSE ($kcal^2/mol^2$)	0.19	0.15	0.18	0.17	0.20	0.22
MAPE (%)	4.81	4.70	5.49	5.72	6.04	5.67
MAE (kcal/mol)	0.33	0.33	0.36	0.37	0.40	0.37
RMSE (kcal/mol)	0.44	0.39	0.43	0.41	0.44	0.46

Table S8: Cross-validation obtained for the MLR models in case of the drugs modified with C_{60}.

**Error**	**Model 1**	**Model 2**	**Model 3**	**Model 4**	**Model 5**	**Model 6**
	**MLR**	**MLR**	**MLR**	**MLR**	**MLR**	**MLR**
MSE ($kcal^2/mol^2$)	0.64	0.53	0.50	0.41	0.55	0.29
MAPE (%)	5.38	5.48	5.17	3.94	5.99	4.56
MAE (kcal/mol)	0.56	0.54	0.51	0.39	0.59	0.45
RMSE (kcal/mol)	0.80	0.73	0.70	0.64	0.74	0.54

Table S9: Cross-validation obtained for the MLR models in case of the drugs modified with C_{60}–COOH.

**Error**	**Model 1**	**Model 2**	**Model 3**	**Model 4**	**Model 5**	**Model 6**
	**MLR**	**MLR**	**MLR**	**MLR**	**MLR**	**MLR**
MSE ($kcal^2/mol^2$)	0.21	1.28	0.96	1.03	0.79	0.58
MAPE (%)	4.06	8.57	6.88	7.84	7.54	5.91
MAE (kcal/mol)	0.38	0.79	0.63	0.73	0.71	0.56
RMSE (kcal/mol)	0.46	1.13	0.98	1.02	0.89	0.76

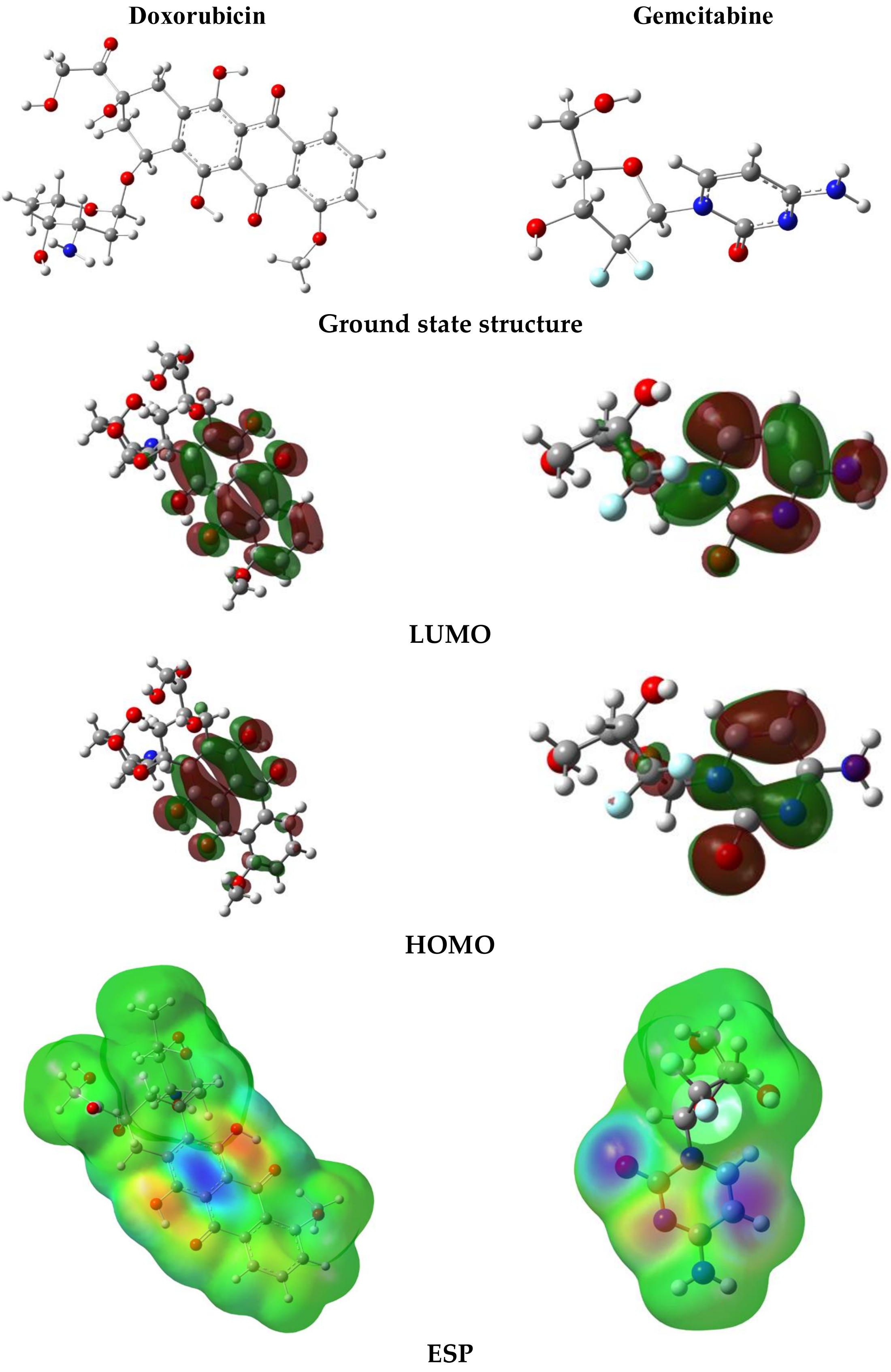

Figure S2: Doxorubicin and gemcitabine molecules in their ground state structures. Also, HOMOs, LUMOs and ESP maps are shown as well.
